# *Chlamydia trachomatis* Co-opts the FGF2 Signaling Pathway to Enhance Infection

**DOI:** 10.1371/journal.ppat.1002285

**Published:** 2011-10-06

**Authors:** Jung Hwa Kim, Shaobo Jiang, Cherilyn A. Elwell, Joanne N. Engel

**Affiliations:** 1 Department of Medicine, University of California San Francisco, San Francisco, California, United States of America; 2 Microbial Pathogenesis and Host Defense Program, University of California San Francisco, San Francisco, California, United States of America; 3 Department of Microbiology and Immunology, University of California San Francisco, San Francisco, California, United States of America; Duke University, United States of America

## Abstract

The molecular details of *Chlamydia trachomatis* binding, entry, and spread are incompletely understood, but heparan sulfate proteoglycans (HSPGs) play a role in the initial binding steps. As cell surface HSPGs facilitate the interactions of many growth factors with their receptors, we investigated the role of HSPG-dependent growth factors in *C. trachomatis* infection. Here, we report a novel finding that Fibroblast Growth Factor 2 (FGF2) is necessary and sufficient to enhance *C. trachomatis* binding to host cells in an HSPG-dependent manner. FGF2 binds directly to elementary bodies (EBs) where it may function as a bridging molecule to facilitate interactions of EBs with the FGF receptor (FGFR) on the cell surface. Upon EB binding, FGFR is activated locally and contributes to bacterial uptake into non-phagocytic cells. We further show that *C. trachomatis* infection stimulates *fgf2* transcription and enhances production and release of FGF2 through a pathway that requires bacterial protein synthesis and activation of the Erk1/2 signaling pathway but that is independent of FGFR activation. Intracellular replication of the bacteria results in host proteosome-mediated degradation of the high molecular weight (HMW) isoforms of FGF2 and increased amounts of the low molecular weight (LMW) isoforms, which are released upon host cell death. Finally, we demonstrate the *in vivo* relevance of these findings by showing that conditioned medium from *C. trachomatis* infected cells is enriched for LMW FGF2, accounting for its ability to enhance *C. trachomatis* infectivity in additional rounds of infection. Together, these results demonstrate that *C. trachomatis* utilizes multiple mechanisms to co-opt the host cell FGF2 pathway to enhance bacterial infection and spread.

## Author Summary

*Chlamydia trachomatis* is an obligate intracellular bacterium that is an important cause of human disease, including sexually transmitted diseases and acquired blindness in developing countries. The inability to carry out conventional genetic manipulations limits our understanding of the mechanisms of *C. trachomatis* binding, entry, and spread. Previous studies have shown that heparan sulfate proteoglycans (HSPGs) play a role in early binding events. As cell surface HSPGs facilitate the interactions of many growth factors with their receptors, we investigated whether HSPG-associated growth factors affect *C. trachomatis* binding or entry. Here, we report the novel finding that Fibroblast Growth Factor 2 (FGF2), a ubiquitously expressed growth factor, enhances *C. trachomatis* binding to host cells in an HSPG-dependent manner. Furthermore, *C. trachomatis* infection stimulates production and release of FGF2 through distinct signaling pathways. Released FGF2 is sufficient to enhance the subsequent rounds of infection. Together, these results demonstrate that *C. trachomatis* utilizes multiple mechanisms to co-opt the host cell FGF2 pathway that sets up a positive feedback loop to enhance bacterial infection and spread.

## Introduction

*Chlamydia trachomatis,* an obligate intracellular bacterium, is the most common bacterial cause of sexually transmitted diseases and non-congenital infertility in Western countries and the leading cause of acquired blindness in developing countries (reviewed in [1]). *C. trachomatis* serovars A–C cause eye disease, serovars D–K cause genital tract infections, and serovars L1–L3 are associated with lymphogranuloma venereum (LGV), a more invasive genital tract disease. *C. pneumoniae* infections result in upper and lower respiratory tract infections and have been linked to a growing number of chronic diseases, including atherosclerosis, multiple sclerosis, and Alzheimer's disease. The capacity of *Chlamydiae* to lead to infertility and blindness, their association with chronic diseases, and the extraordinary prevalence and array of these infections make them public concerns of primary importance.

All *Chlamydia* species share a dimorphic life cycle in which they alternate between an extracellular, spore-like form, the elementary body (EB), and an intracellular, metabolically active but non-infectious form, the reticulate body (RB) [2]. *Chlamydiae* can productively infect most cultured cells, suggesting that the receptor(s) is widespread and/or that there are multiple receptors. Although neither the bacterial ligand nor the host receptor(s) have been definitively identified, it is thought that binding is a two step process that involves an initial reversible interaction between the EB and the host cell followed by high affinity irreversible binding to a secondary receptor (reviewed in [3]).

After binding, *Chlamydiae* induce their uptake into non-phagocytic cells through small-GTPase dependent reorganization of the actin cytoskeleton to form microvillus-like structures (reviewed in [4]). Both bacterial and host factors are implicated in this process. These include (i) translocated actin recruiting phosphoprotein (TARP), a chlamydial protein secreted into the host cell cytosol upon bacterial binding, (ii) the host tyrosine kinases platelet-derived growth factor receptor (PDGFR)-β and Abl kinase, and (iii) other host actin cytoskeleton regulatory proteins that are recruited to TARP and/or to PDGFR [5], [6], [7]. Following entry, EBs are sequestered within a membrane-bound compartment, termed a vacuole or inclusion, which quickly dissociates from the endo-lysosomal pathway and avoids fusion with phagosomes (reviewed in [8]). Subsequently, EBs differentiate into RBs, which replicate by binary fission within the enlarging inclusion over a 12–72 hr time period. In response to an as yet unknown signal, RBs redifferentiate into EBs and are released from the host cell through cell lysis or active extrusion [9], where they can initiate secondary rounds of infection in neighboring cells. *Chlamydia* infection alters the transcription of many host genes, including pro-inflammatory cytokines, and regulators of apoptosis, cell differentiation, and the cytoskeleton [10], [11]. The combination of tissue destruction and host inflammatory responses is at least partially responsible for the devastating long-term consequences of *Chlamydia* infection [12].

For many *C*. *trachomatis* serovars, the initial reversible binding step is thought to involve binding to host heparan sulfate proteoglycans (HSPGs), as addition of excess heparin or heparan sulfate but not chondroitin sulfate, inhibits binding [13], [14], [15]. HSPGs are extraordinarily heterogeneous structures that are composed of a linear array of repeating dissacharides covalently linked to various core proteins and are variably sulfated [16]. A large number of growth factors, cytokines, and differentiation factors, as well as various classes of cell surface receptors, extracellular matrix proteins, and enzymes, bind to HSPGs [17]. Prominent among these is fibroblast growth factor 2 (FGF2; also known as basic fibroblast growth factor), one of the 23 FGF family members.

FGF2 is critical during development and can also mediate many cellular responses in adult tissues by binding to and activating the receptor tyrosine kinases FGFR1-FGFR4 [18]. FGF2 itself is heterogeneous; it is expressed as five different isoforms (34, 24, 22.5, 22, and 18 kDa isoforms) that result from alternative translational start sites within a single mRNA. The 18 kDa isoform can be further processed into a 16 kDa protein, which has identical properties [19]. Only the 16/18 kDa isoforms are secreted, where they bind to cell surface HSPGs, functioning in a paracrine and autocrine manner to activate FGF receptor (FGFR) family members. In contrast, the larger FGF2 isoforms, translated from non-canonical CUG start codons, have nuclear localization signals and are found primarily in the nucleus; their functions remain incompletely elucidated [20], [21]. Although FGF2 dimers can bind to and activate FGFR, binding of HSPGs stabilizes activated ligand-bound dimeric FGFR [22], [23], [24]. FGFR activation leads to recruitment and tyrosine phosphorylation of the docking proteins FGFR substrate (FRS) 2α and FRS2β, as well as SHC1, followed by recruitment and activation of Grb2, SOS, Ras, mitogen-activated protein kinase (MAPK), and phosphatidyl inositol-3 kinase (PI3K) [25], [26].

In this work, we examined the effect of HSPG-associated growth factors on *C. trachomatis* infection. We report that *C. trachomatis* serovar L2 EBs bind to FGF2, which facilitates bacterial binding, and that FGF2 promotes internalization of EBs via FGFR. In addition, *C. trachomatis* stimulates FGF2 transcription, production, and release to facilitate additional rounds of infection. Upregulation of FGF2 is independent of FGFR activation but involves bacterial protein synthesis and activation of the extracellular signal-regulated kinase (Erk) 1/2 pathway. Many of these findings were also observed with *C. trachomatis* serovar E, whose binding was also enhanced by FGF2 in an HSPG-dependent manner. Thus, *C. trachomatis* utilizes multiple mechanisms to co-opt the host cell FGF2 pathway to enhance bacterial infection and spread.

## Results

### FGF2 is sufficient to stimulate *C. trachomatis* binding and vacuole formation

**Figure 1:**
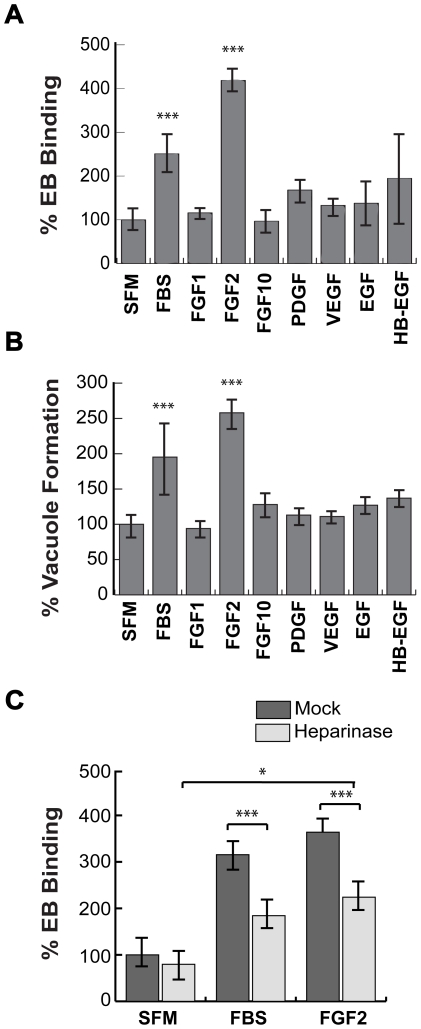
FGF2 stimulates *C. trachomatis* L2 binding in an HSPG-dependent manner. (A, B) HeLa cells were serum starved for 2 hr and then infected with *C. trachomatis* L2 in SFM, in FBS-containing media, or in SFM supplemented with the indicated growth factors (100 ng/mL) for 1 hr. Binding and vacuole formation was quantified at 1 hpi (A) and 20 hpi (B), respectively. Shown is the mean ± SEM of three independent experiments normalized to SFM. *** p<0.001 (C) HeLa cells were treated with heparinase for 2 hr in SFM and infected for 1 hr with *C. trachomatis* L2 either in SFM, in FBS-containing media, or in SFM supplemented with FGF2 (100 ng/mL). Shown is the mean binding ± SEM normalized to mock-treated cells infected in SFM from four independent experiments. * p<0.05, *** p<0.001

Given the previously published involvement of HSPGs on initial steps in *C. trachomatis* binding [5], [27], we tested whether HSPG-dependent growth factors enhance *C. trachomatis* binding to cultured cervical cells. HeLa cells were serum-starved for 2 hrs and then infected with *C. trachomatis* serovar L2 in serum-free media (SFM), in the presence of 10% fetal bovine serum (FBS), or HSPG-dependent growth factors. Binding and vacuole formation were measured at 1 hr post-infection (hpi) and 20 hpi, respectively. Addition of 10% FBS or purified FGF2 stimulated binding and vacuole formation ∼2–3 fold (Figs 1A–C). In contrast, neither PDGF-BB, FGF-1, FGF-10, Vascular Endothelial Growth Factor (VEGF), Epidermal Growth Factor (EGF), nor heparin-binding EGF-like growth factor (HB-EGF) significantly enhanced EB binding or vacuole formation (Figs 1A, B). FGF2 increased *C. trachomatis* binding in a dose-dependent, saturable manner, with half-maximal binding observed at ∼50 ng/mL of FGF2 (Fig S1A). We also observed that FGF2 was sufficient to enhance *C. trachomatis* vacuole formation in H292 cells, a human lung epithelial cell line (Fig S1B).

As FGF2-FGFR interactions are stabilized through binding to HSPGs, we tested whether FGF2-stimulated *C. trachomatis* binding is HSPG-dependent (Fig 1C). HeLa cells were treated with heparinase for 2 hrs in SFM. After washing to remove the heparinase, the cells were infected for 1 hr with *C. trachomatis* in the presence or absence of FGF2 or serum. Pre-treatment of cells with heparinase significantly decreased FBS- or FGF2-stimulated binding (p<0.005), but had no statistically significant effect on EB binding in SFM, ruling out non-specific effects (Fig 1C). In control experiments, we verified that heparinase-treatment was effective, as surface staining with an anti-heparan sulfate antibody was decreased by 60% under the conditions of our experiments (Fig S2). The residual stimulatory effect of FGF2 after heparinase treatment may reflect incomplete enzymatic removal or may represent HSPG-independent FGF2 stimulation. Nonetheless, these results show that FGF2 is sufficient to enhance *C. trachomatis* binding in an HSPG-sensitive manner and that FGF2-mediated *C. trachomatis* binding leads to productive infection.

### FGF2 binds directly to EBs and facilitates binding to HeLa cells

**Figure 2:**
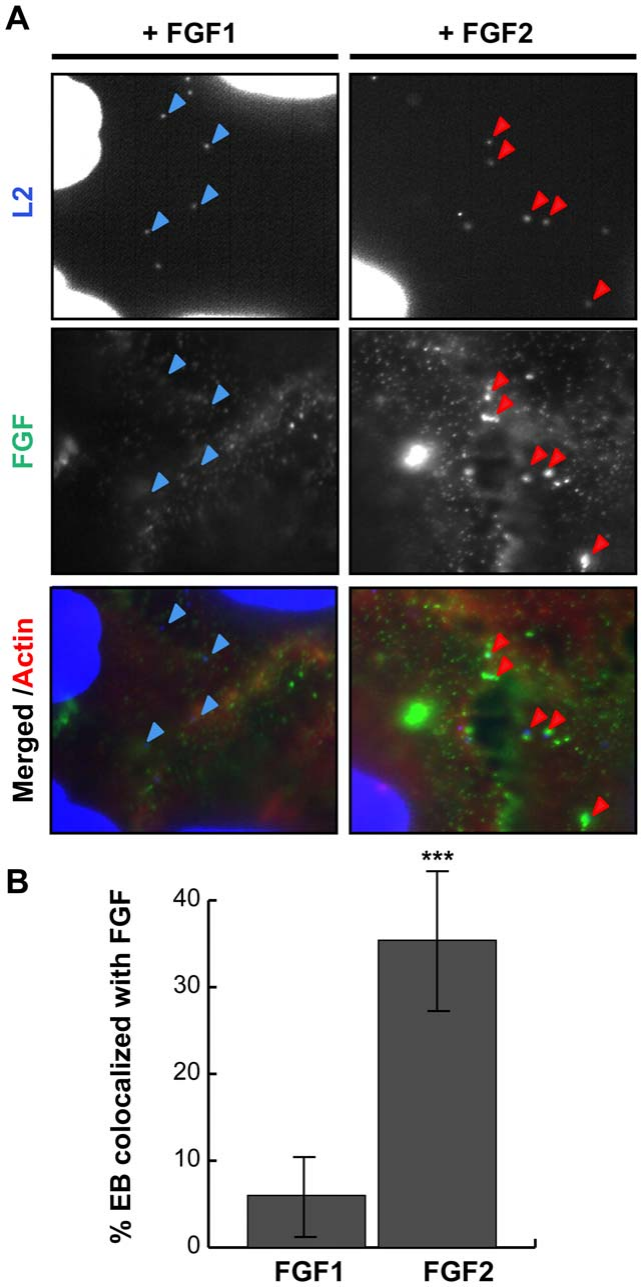
FGF2 co-localizes with cell surface bound EBs. HeLa cells were infected for 1 hr with *C. trachomatis* L2 in SFM supplemented with FGF1 (left panels; 100 ng/mL) or with FGF2 (right panels; 100 ng/mL). (A) Cells were fixed and stained with FGF1 antibody (left panels) or FGF2 antibody (right panels). Cell surface associated EBs and host cell nuclei were visualized by DAPI (blue) staining. Arrows point to EBs. (B) Co-localization of EBs with FGF1 or FGF2 was quantified from at least 8 different fields. The data are expressed as a mean percentage of FGF-associated bacteria (± SEM) compared to total bacteria. *** p<0.001

As FGF2 is known to bind to both FGFR and to HSPGs on the cell surface, it is possible that FGF2 could facilitate EB binding through direct interactions with *C. trachomatis* and with FGFR, thereby functioning as a bridging molecule to bring EBs to the host cell surface. Alternatively, FGF2 could indirectly enhance *C. trachomatis* binding by upregulating a host cell receptor or stimulating its endocytosis as a consequence of FGFR activation. The first model predicts that FGF2 would co-localize with cell surface bound EBs, whereas in the second model, FGF2 binding could be spatially distinct from EB binding. We therefore examined whether FGF2 colocalizes with *C. trachomatis* at the cell surface. HeLa cells were serum starved and infected with *C. trachomatis* for 45 min in the presence of FGF2, or as a negative control, with FGF1, and co-localization of EBs with FGF1 or FGF2 was quantified by immunofluorescence (IF) microscopy. As shown in Fig 2, ∼35% of surface bound EBs colocalized with FGF2, whereas only ∼5% of surface bound EBs were found in association with FGF1 (p<0.001).

**Figure 3:**
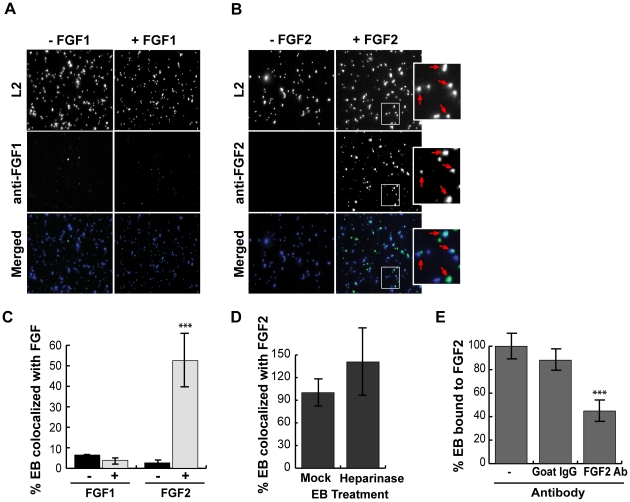
FGF2 binds to renograffin-purified *C. trachomatis* L2 EBs. Renograffin-purified EBs were incubated with 100 ng/mL of FGF1 (A) or FGF2 (B) in SFM containing 0.1% BSA for 1 hr at 37°C. The EB-FGF mixture was centrifuged onto coverslips, fixed, and stained with DAPI (to visualize the EBs) and FGF1 antibody (A) or FGF2 antibody (B). Red arrows in the enlarged images show co-localization of FGF2 with EBs. (C) Co-localization of EBs with FGF1 or FGF2 was quantified from at least 8 different fields. The data are expressed as a mean percentage of FGF-associated bacteria (± SEM) compared to total bacteria. *** p<0.001 compared to the absence of growth factor (D) EBs that had been either mock- or heparinase-treated for 2 hrs were incubated with FGF2 as described in 3A. Co-localization of EBs with FGF2 was quantified as described in 3C. The values are expressed as a mean percentage (± SEM) of FGF2-associated bacteria compared to total bacteria from three independent experiments. The data are normalized to the mock treated EBs. The difference in FGF2 binding between mock- and heparinase-treated EBs was not statistically significant (P>0.05) (E) EBs and FGF2 were incubated in the presence of isotype matched Goat IgG or FGF2 neutralizing antibody for 1 hr. The FGF2-antibody mixture was centrifuged onto coverslips, fixed, and stained. The values are expressed as a mean percentage (± SEM) of FGF2-associated bacteria compared to total bacteria. The data are normalized to the no antibody control. *** p<0.001

We next examined whether purified growth factors could bind directly to EBs in vitro. Renograffin-purified EBs were incubated with FGF2 or FGF1 in SFM at 37°C for 1 hr, centrifuged onto coverslips, fixed, stained with antibodies to FGF1 or FGF2, and examined by IF. In the absence of either growth factor, very little antibody to FGF1 or FGF2 bound to purified EBs (Fig 3A and B). Surprisingly, addition of purified FGF2 from two different sources increased the frequency of anti-FGF2 staining of EBs from less than 10% to 50% (Fig 3C and data not shown; p<0.001). In contrast, only ∼5% of FGF1 associated with EBs, and this fraction was not enhanced upon addition of exogenous FGF1 (Figs 3A and 3C). In control experiments, anti-FGF1 antibody recognized FGF1 bound to the cell surface (Fig 2A). Co-localization of FGF2 with purified EBs was not diminished by pre-treatment of EBs with heparinase (Fig 3D, P>0.05).

As a further test of specificity, we examined whether neutralizing antibodies against FGF2 decreased FGF2 binding to EBs. EBs were incubated with SFM containing 100 ng/mL FGF2 in the presence of the indicated neutralizing antibody for 1 hr and then examined for FGF2 binding to EBs by IF. As shown in Fig 3E, addition of FGF2 antibodies decreased FGF2 binding to EBs by approximately 2-fold compared to incubation with an isotype-matched control antibody or in the absence of antibody (p<0.001). Together, these results suggest that EBs can bind specifically to FGF2 to facilitate binding to the surface of host cells. We note that these experiments do not eliminate the possibility that FGF2 may also stimulate *C. trachomatis* binding through additional mechanisms.

### Cell associated FGF2 contributes to *C. trachomatis* binding

**Figure 4:**
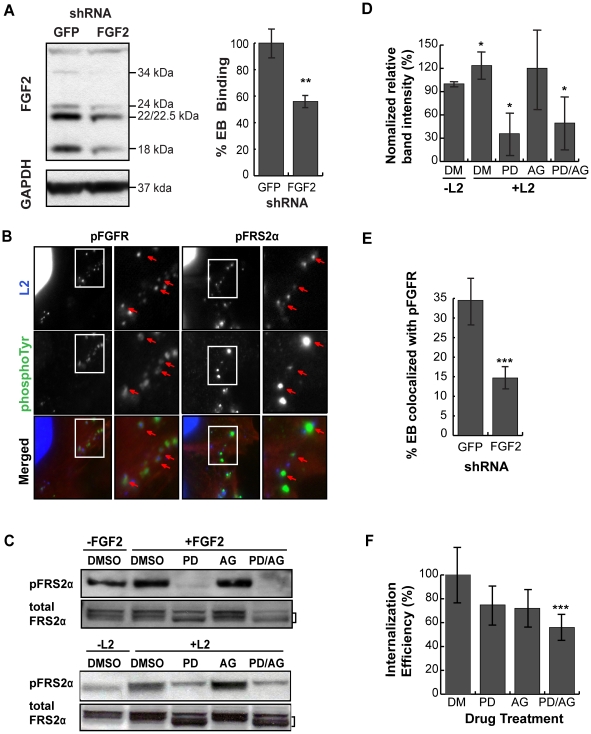
Cell associated FGF2 contributes to *C. trachomatis* L2 binding, activation and recruitment of FGFR/FRS2α, and bacterial entry. (A) Left panel; HeLa cells were transfected with shRNA to GFP (control) or FGF2. After 72 hr, cell lysates were immunoblotted with anti-FGF2 or anti-GAPDH (control) to assess efficiency of depletion. The molecular weights of the five FGF2 isoforms (upper panel) and GAPDH (lower panel) are marked. Right panel; HeLa cells transfected with GFP or FGF2 shRNA were infected with *C. trachomatis* L2 in SFM and binding was measured at 1 hpi. Shown is the mean binding ± SEM compared to GFP shRNA treated cells from three independent experiments. **p<0.01 (B) HeLa cells that were serum-starved for 2 hr were infected with *C. trachomatis* L2 for 45 min. Cells were fixed and stained with antibodies to phospho-FGFR (pFGFR; green) or to phospho-FRS2α (pFRS2α; green). EBs and host cell nuclei were visualized with DAPI (blue). Enlargements of the boxed areas are shown to the right. Red arrows point to EBs. (C) HeLa cells were incubated in SFM with DMSO, FGFR inhibitor PD173074 (200 nM), PDGFR inhibitor AG1296 (10 µM), or both inhibitors for 2 hr. Samples were either stimulated with FGF2 (50 ng/mL) for 5 min (upper panel) or infected with *C. trachomatis* L2 for 45 min (lower panel) in the presence of the indicated drug. Lysates were immunoblotted with antibodies to pFRS2α or to FRS2α. FRS2α migrates as multiple bands, delineated by the bracket. Immunoblots are representative of three independent experiments. (D) The percentage of pFRS2α compared to total FRS2α was quantified using densitometry analysis of the immunoblots (Fig 4C) and normalized to DMSO (DM) treated uninfected samples. The values represent the mean (± SEM) of three independent experiments. *p<0.05 compared to DMSO treated uninfected cells. (E) The effect of FGF2 depletion by shRNA on co-localization of pFGFR with surface bound EBs was quantified in at least 8 different fields. Shown are the average percent of EBs that co-localized with pFGFR (± SEM). ***p<0.001 (F) FGFR and PDGFR have redundant roles in *C. trachomatis* L2 entry. HeLa cells pre-incubated in SFM with DMSO, PD173074, AG1296, or both inhibitors for 2 hr were infected with *C. trachomatis* L2 for 1 hr in the presence of drug in SFM. Cells were fixed and analyzed by inside-out staining to distinguish between total cell associated bacterial and internalized bacteria as described in Materials and Methods. The internalization efficiency (the percentage of internalized EBs/total cell associated EBs) in each case was normalized to DMSO (DM) treated cells. Shown is the mean (± SEM) of three independent experiments. ***p<0.001 compared to DMSO treated cells

The ability of exogenous FGF2 to stimulate *C. trachomatis* binding led us to examine whether endogenous FGF2 contributed to *C. trachomatis* binding. Since FGF2 is expressed as 5 different isoforms that result from alternative translation initiation sites (not from differential splicing), we used a short hairpin RNA (shRNA) directed against a region common to all isoforms. Cells were transfected with an shRNA against FGF2 or against GFP (control) (Fig 4A, left panel). Infections were performed in SFM, as the presence of exogenous FGF2 in serum would likely mask the effects of RNAi depletion. Under conditions where total FGF2 was depleted by 60% as determined by densitometry, we observed a 40% decrease in *C. trachomatis* binding compared to cells treated with a control (GFP) shRNA (p<0.01; Fig 4A, right panel). These results suggest that endogenous FGF2 contributes to *C. trachomatis* binding.

### FGFR is activated upon infection and recruited to cell associated *C. trachomatis*

Our results predict that FGF2-mediated binding of EBs to the host cell surface should result in activation of FGFR and downstream signaling pathways. FGF2 binds to and activates most isoforms of FGFR (FGFR1 IIIb and IIIc, FGFR2 IIIc, FGFR3 IIIc, and FGFR4)[28], [29], [30], resulting in activation of their tyrosine kinase activity, autophosphorylation of tyrosine 653/654, and phosphorylation of the scaffolding protein, FRS2α, which is constitutively associated with FGFR. Upon tyrosine phosphorylation, FRS2α functions as a site for coordinated assembly of a multi-protein signaling complex, including Grb and Shp2, leading to activation of the PI3K and the Ras/Erk pathways [26].

We used several approaches to determine whether FGFR is activated upon *C. trachomatis* binding. First, we examined by IF microscopy whether activated FGFR (pFGFR) co-localized with cell surface bound EBs, using a monoclonal antibody that specifically recognizes phosphorylation of the conserved tyrosines at 653/654. IF analysis revealed that pFGFR was found with cell-associated EBs (Fig 4B). We were unable to detect changes in the localization of total FGFR1 or FGFR2 (data not shown), possibly because only a small fraction of total cellular FGFR is recruited and phosphorylated at the site of EB binding. Second, since activation of FGFR is required for FRS2α phosphorylation, we used IF microscopy to determine whether phosphorylated FRS2α is recruited to the site of *C. trachomatis* binding. Indeed, an antibody directed against phosphotyrosine 436 of FRS2α (pFRS2α) co-localized with cell-surface bound EBs (Fig 4B, right panels). Third, we measured activation of FRS2α by immunoblotting with an antibody to pFRS2α. At 45 min pi, increased phospho-FRS2α was detected in *C. trachomatis*-infected HeLa cells (Fig 4C, lower panel), at levels similar to what is observed at 5 min after addition of FGF2 to serum starved HeLa cells (Fig 4C, upper panel). *C. trachomatis*-induced FRS2α phosphorylation was blocked by the FGFR inhibitor, PD173074 (Figs 4C and 4D), confirming that FRS2α phosphorylation was a result of *C. trachomatis*-activation of FGFR. Depletion of endogenous FGF2 by shRNA reduced the percent of EBs colocalized with pFGFR from 35% to 15%, suggesting that recruitment of pFGFR to the site of *C. trachomatis* binding is FGF2-dependent (Fig 4E). Together, these findings indicate that FGF2 mediated *C. trachomatis* binding is associated with the activation and recruitment of phosphorylated FGFR and FRS2α.

### FGFR and PDGFR have redundant roles in *C. trachomatis* entry

There is evidence of cross-talk between growth factor receptors; for example, PDGFR has been shown to activate FGFR [31]. Since we recently reported that PDGFR contributes to *C. trachomatis* binding and internalization [5], we examined the possible interrelationship between FGFR and PDGFR signaling pathways during *C. trachomatis* infection in SFM using pharmacological inhibitors. In control experiments, we established that AG1296, a PDGFR inhibitor, blocked PDGF-dependent PDGFR activation (data not shown) but had no effect on FGF2 dependent activation of FRS2α (Fig 4C, upper panel). Importantly, AG1296 did not inhibit *C. trachomatis*-induced FRS2α activation (Figs 4C, lower panel, and 4D). These experiments confirm that FGFR activation during *C. trachomatis* infection is independent of PDGFR activation.

Using PD173074 or AG1296 singly or in combination, we examined the effect of blocking FGFR or PDGFR activation on the efficiency of EB internalization. HeLa cells were pre-incubated in SFM containing PD173074, AG1296, or both drugs for 2 hrs, and then infected with *C. trachomatis* in SFM in the presence of drug(s). At 1 hpi, the bacterial internalization efficiency (the fraction of bound EBs that were internalized) was quantified by inside-out staining as described in Materials and Methods. Treatment with either drug alone showed a small effect on internalization that failed to reach statistical significance. However, treatment with both drugs showed an additive effect, decreasing internalization by 40% (p<0.001; Fig 4F). This result indicates that bacterial entry occurs through redundant pathways that involve activation of PDGFR and FGFR.

### *C. trachomatis* infection stimulates FGF2 expression, production, and release from HeLa cells

**Figure 5:**
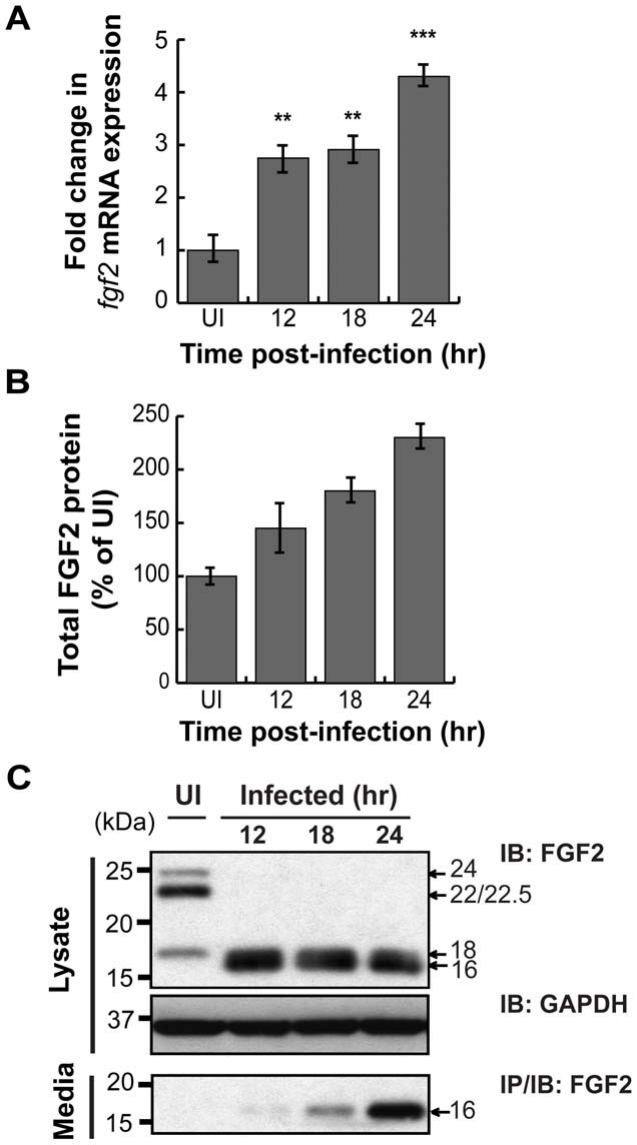
*C. trachomatis* L2 infection stimulates FGF2 expression, production, and release. (A) Total RNA was isolated from the HeLa cells infected with *C. trachomatis* L2 for indicated time. *fgf2* mRNA was assessed by qRT-PCR and normalized to *gapdh* mRNA. (B) Total FGF2 protein (cell associated and secreted) was measured by Quantikine kit and normalized to uninfected cells (UI) and normalized for total cell number by quantifying total LDH activity. (C) Cell lysates (upper panel) and cell supernatants (lower panel) of FGF2 were collected from HeLa cells infected with *C. trachomatis* L2 for the indicated times. FGF2 was detected by immunoblot (upper panel) or by immunoprecipitation (lower panel). GAPDH serves as a loading control.

**Figure 6:**
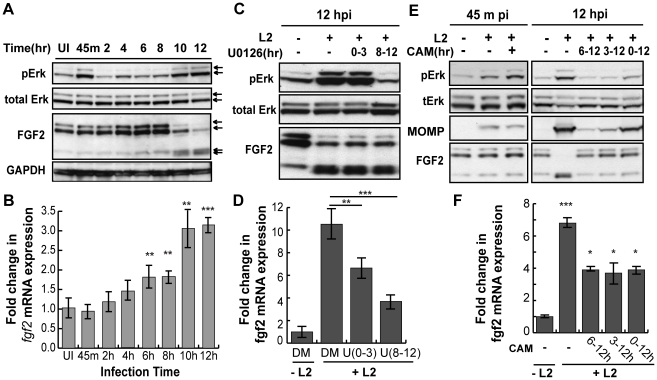
*C. trachomatis* L2 induces a biphasic activation of Erk1/2 which contributes to the induction of *fgf2* mRNA expression. (A) Upper 2 panels: Erk1/2 activation in HeLa cells infected with *C. trachomatis* L2 for the indicated times was examined by immunoblotting cell lyates with antibodies to phospho-Erk (pERK) and to total Erk. Arrows indicate Erk1/2 (p42/p44). Two peaks of Erk1/2 activation are noted, at 45 min pi and at 10–12 hpi. Lower 2 panels: The same lysates from upper panel were immunoblotted with antibodies to FGF2 or to GAPDH (loading control). In the FGF2 immunoblot, arrows indicate the 24, 22.5/22, 18, and 16 kDa isoforms of FGF2. An abrupt change in FGF2 isoforms is noted at 10 hpi. (B) Total mRNA was isolated from HeLa cells infected with *C. trachomatis* L2 for the indicated times and the fold change in *fgf2* mRNA relative to *gapdh* mRNA was measured by qRT-PCR. An increase in *fgf2* mRNA is detectable by 6 hpi and increases further at 10–12 hpi. **p<0.01, ***p<0.001, compared to uninfected (UI) samples. (C, D) HeLa cells were infected with *C. trachomatis* L2 for 12 hrs in the presence of 10 µM U0126 (U) between 0–3 hpi or 8–12 hpi. Lysates were immunoblotted with antibodies to pErk or to total Erk. The fold change in *fgf2* mRNA relative to *gapdh* mRNA was quantified by qRT-PCR using total RNA isolated at 12 hpi. Inhibition of either the early peak of Erk activation or the late peak of Erk activation decreased *C. trachomatis* L2 induction of *fgf2* mRNA but did not affect the distribution of FGF2 isoforms. **p<0.01, ***p<0.001 compared to *C. trachomatis* L2 infected cells. (E, F) HeLa cells were infected with *C. trachomatis* L2 for 45 min or 12 hrs in the absence or presence of chlorampenicol (CAM; 100 µg/mL) for the indicated times. (E) Erk activation and FGF2 isoforms were detected by immunoblotting cell lyates with the indicated antibody. MOMP serves as a control for bacterial replication. (F) Total RNA was isolated at 12 hpi and the fold change in *fgf2* mRNA relative to *gapdh* mRNA was monitored by qRT-PCR. Bacterial protein synthesis is required for Erk activation and for the change in FGF2 isoforms. *p<0.05, ***p<0.001 compared to uninfected (UI) cells.

As host microarray analyses previously revealed that *C. pneumoniae* induces *fgf2* transcription [32], [33], we tested whether *C. trachomatis* induces *fgf*2 transcription. We performed qRT-PCR with *fgf2* specific primers and found that *C. trachomatis* infection increased *fgf2* mRNA expression 3-fold relative to *gapdh* at 12 hpi and 4–5 fold at 24 hpi (Fig 5A); in contrast, no significant increase in *fgf1* expression was detected (Fig S3). Likewise, total FGF2 (secreted and cell associated; measured by ELISA assay) increased throughout infection (Fig 5B). The increased FGF2 transcription and production was accompanied by a striking change by 12 hpi in the distribution of the FGF2 isoforms (Figs 5C and 6A). The 22 kDa, 22.5 kDa, and 24 kDa forms became undetectable in cell lysates by immunoblot analysis, whereas the 18 kDa form and a slightly faster migrating form (which likely corresponds to a previously described 16 kDa secreted form that has similar biological activities to the 18 kDa form [19]) appeared to increase in the cell-associated fraction. By 18 hpi, a corresponding increase in the 16/18 kDa isoforms was detected in the culture medium (Fig 5C, lower panel).

### *C. trachomatis* induction of *fgf2* transcription is mediated by the Erk1/2 signaling pathway

Diverse stimuli induce *fgf2* transcription, including FGF-mediated activation of FGFR [34] and Erk1/2 activation [35]. As *C. trachomatis* infection has been shown to activate Erk1/2 [36], [37], [38], [39], [40], [41], we tested the role of Erk1/2 in upregulation of *fgf2* transcription. When the kinetics of *C. trachomatis*-induced Erk1/2 activation were examined by immunoblot analysis with an antibody that specifically recognizes the phosphorylated (i.e. activated) form of Erk1/2, we detected an early peak of Erk1/2 phosphorylation at 45 min pi followed by a second peak of Erk1/2 phosphorylation beginning at 10 hpi (Fig 6A), consistent with previously reported results [36], [42]. We performed a more detailed time course of the induction of *fgf2* mRNA and found a small but statistically significant (p<0.05) enhancement of *fgf2* transcription as early as 6–8 hpi, which further increased at 10 hpi (Fig 6B). Thus, the kinetics of *C. trachomatis*-induced upregulation of *fgf2* transcription is consistent with the involvement of Erk1/2 signaling.

To determine whether Erk1/2 activation is necessary for up-regulation of *fgf2* transcription in response to *C. trachomatis* infection, we monitored the effect of inhibiting Erk1/2 on *fgf2* transcription during infection (Fig 6C and D). The Erk/MAPK kinase (MEK) inhibitor U0126 was added for the first 3 hrs of infection (0–3 hpi; to prevent the first wave of Erk1/2 activation) or for the last 4 hrs of infection (8–12 hpi; to prevent the second wave of Erk1/2 activation), and *fgf2* transcription was assessed at 12 hpi. Inhibition of early Erk1/2 activation resulted in an approximately 30% reduction in *fgf2* mRNA levels at 12 hpi, while blocking late Erk1/2 activation decreased *fgf2* mRNA levels approximately 60%. These results suggest that both the early and late peaks of Erk1/2 activation contribute to the upregulation of *fgf2* mRNA observed at 12 hpi. The reduction in *fgf2* transcription by U0126 was not an indirect consequence of blocking early events in the life cycle, as short-term treatment with U0126 had no effect on *C. trachomatis* binding, uptake, or replication (Figs S4A–C). We noted that the partial decrease in *fgf2* transcription in the presence of the MAPK inhibitor (Fig 6D) was not immediately reflected in decreased levels of cell associated FGF2 (Fig 6C). Post-transcriptional regulation could account for this observation [43], [44], [45], suggesting that regulation of FGF2 is complex and subject to multiple levels of control.

Since Erk1/2 activation was previously reported to be dependent upon *C. trachomatis* replication [36], we examined whether bacterial protein synthesis was required for the early and/or late wave(s) of Erk1/2 activation. The addition of the bacterial protein synthesis inhibitor chloramphenicol (CAM) did not reduce Erk1/2 phosphorylation at 45 min pi, suggesting that bacterial protein synthesis is not required for the early peak of Erk activation (Fig 6E, left panel). In contrast, addition of CAM to *C. trachomatis*-infected cells from 0–12 hpi, 3–12 hpi, or 6–12 hpi abrogated late Erk1/2 activation, as assessed at 12 hpi (Fig 6E, right panel). Consistent with its role in late Erk1/2 activation, inhibition of bacterial protein synthesis partially reduced the increase in *fgf2* transcription at 12 hpi (Fig 6F).

Although FGFR or PDGFR activation are known to activate Erk1/2 and to enhance *fgf2* transcription [46], [47], [48], pharmacologic inhibition of these growth factor receptors during *C. trachomatis* infection only partially blocked Erk1/2 activation at 45 min pi (Fig S5A), did not inhibit Erk1/2 activation at 12 hpi (data not shown), and had no statistically significant inhibitory effect on *C. trachomatis*-mediated induction of *fgf2* transcription (Fig S5B). Together, these results suggest that both waves of *C. trachomatis*-mediated Erk1/2 activation contribute to the induction of *fgf2* transcription and that this transcriptional regulation is independent of FGFR receptor activation.

### High molecular weight (HMW) FGF2 isoforms are degraded during intracellular *C. trachomatis* replication

**Figure 7:**
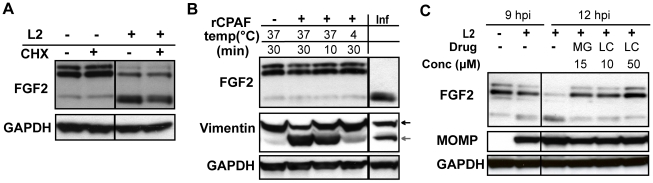
HMW FGF2 isoforms are degraded by *C. trachomatis* L2-induced host proteases. (A) HeLa cells were infected with *C. trachomatis* L2 for 12 hr. For the indicated samples, cycloheximide (CHX; 100 µM) was present from 8–12 hpi. Lysates were immunoblotted with an antibody to FGF2 or GAPDH (loading control). (B) Hela cell lysates were incubated with recombinant CPAF under the indicated conditions and the distribution of FGF2 isoforms was assessed by immunoblotting with an antibody to FGF2. For comparison, the distribution of FGF2 isoforms in *C. trachomatis* L2-infected HeLa cells (Inf) at 12 hpi is shown in the right lane. Vimentin serves as a positive control for CPAF activity. GAPDH serves as a loading control. (C) HeLa cells were infected with *C. trachomatis* L2 for 12 hrs and treated either with DMSO, MG132 (MG; 15 µM) or Lactacystin (LC; 10 µM or 50 µM) from 9–12 hpi. Cell lysates were collected at 9 hpi or 12 hpi as indicated and immunoblotted with antibodies to FGF2, MOMP, (control for bacterial replication), or GAPDH (loading control).

We examined whether Erk1/2 activation, bacterial protein synthesis, and/or host protein synthesis is required for the change in FGF2 isoforms. Addition of U0126 to *C. trachomatis*-infected cells from 0–3 hpi or from 8–12 hpi had no effect on the change in FGF2 isoforms (Fig 6C, lower panel). However, addition of CAM from 0–12 hpi or from 6–12 hpi prevented the change in FGF2 isoforms, suggesting that either de novo synthesis of a bacterial protein or bacterial growth was required for this FGF2 isoform change (Fig 6E, right panel). Furthermore, the eukaryotic translation inhibitor cycloheximide (CHX) did not prevent the *C. trachomatis*-induced changes in FGF2 isoforms (Fig 7A), indicating that these changes occurred at a post-translational step.

The loss of the 22 kDa, 22.5 kDa, and 24 kDa isoforms could arise through host-mediated degradation or through a secreted chlamydial protease, such as Chlamydial Protease/proteasome-like factor (CPAF), a broad spectrum protease [49]. We first tested whether recombinant CPAF could alter the FGF2 isoform distribution by incubating recombinant CPAF with uninfected HeLa cell lysates. No change in the FGF2 isoform distribution was observed (Fig 7B), although CPAF was active against its known substrate vimentin (Fig 7B)[50].

We next determined whether host proteosomal enzymes might be involved in degradation of HMW FGF2 isoforms. Treatment of cells from 9–12 hpi with the proteosome inhibitors Lactacystin or MG132 prevented the change in FGF2 isoforms (Fig 7C). Together, these results suggest that the abrupt change in FGF2 isoforms is independent of Erk1/2 activation, does not involve CPAF, and likely involves degradation by host proteosomal proteases activated during *C. trachomatis* intracellular replication. It is also possible that an as yet to be identified *C. trachomatis* protease that is secreted into the host cell cytosol and that is inhibited by Lactacystin or MG132 is responsible for the loss of the HMW FGF2 isoforms.

### *C. trachomatis* induced secretion of FGF2 is caspase-1 independent and may accompany host cell lysis

We were particularly interested in our observation that *C. trachomatis* infection increases the secretion of the 16/18 kDa FGF2 isoforms, as these isoforms are important for FGFR activation and for *C. trachomatis* binding. These isoforms lack a canonical secretion signal, and their secretion does not involve the classical ER-Golgi secretion pathway [51], [52]. The mechanism of FGF2 secretion is incompletely understood; there are reports that FGF2 release is associated with membrane blebs, caspase-1 activation, and/or the Na-K ATPase [53], [54], [55]. Recent studies reported that FGF2 can be directly translocated across the plasma membrane in a process which depends on the ability of FGF2 to bind HSPGs and a transient interaction between FGF2 and phosphatidylinositol-4,5-bisphosphate (PI(4,5)P_2_) [21].

As *C. trachomatis* has been shown to induce caspase-1 activation [56], we tested whether caspase-1 is involved in the *C. trachomatis* mediated release of FGF2. *C. trachomatis* infected cells were stained with an antibody to p20, one of the two cleavage products of activated caspase-1. An increase in p20-positive cells was not detected until 18 hpi and became most marked at 24 hpi (Fig S6A), consistent with previously published studies showing that *C. trachomatis* activation of caspase-1 is a late event in the intracellular life cycle [56]. Thus, activation of caspase-1 does not correlate with *C. trachomatis*-induced FGF2 release seen from 12 hpi. Likewise, neither of the caspase-1 inhibitors YVAD nor WEHD altered production (data not shown) or release (Fig S6B) of FGF2 during *C. trachomatis* infection. Although our studies do not rule out other active pathways of release, they suggest that the *C. trachomatis*-induced release of the FGF2 is likely independent of caspase-1 activation.

**Figure 8:**
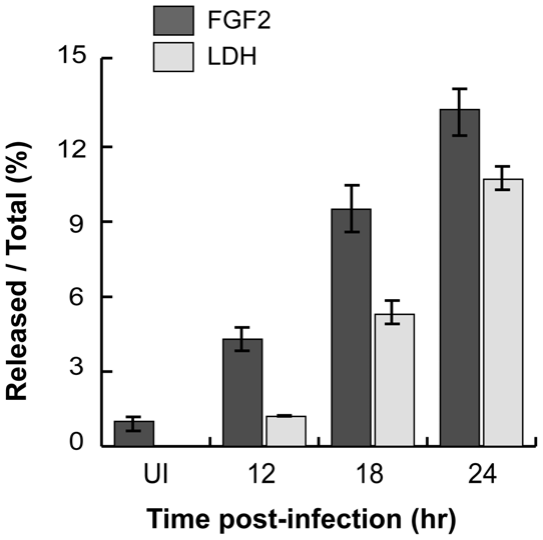
*C. trachomatis* L2-induced FGF2 release correlates with cell lysis. FGF2 or LDH in the cell lysate or in the supernatant was measured by FGF2 Quantikine kit or the Cytotox 96 non-radioactive cytotoxicity assay kit, respectively. Shown is the percentage of FGF2 (black bars) or LDH (grey bars) in the supernatant compared to total.

As *C. trachomatis* infection ultimately leads to host cell death and lysis [2], we considered the possibility that *C. trachomatis*-induced FGF2 release may be a consequence of host cell lysis. As shown in Fig 8, the kinetics of FGF2 secretion in *C. trachomatis*-infected cells correlates with and is proportional to host cell lysis, as determined by LDH release. Together, these results suggest that one mechanism by which FGF2 may be released from *C. trachomatis*-infected cells is by passive release secondary to *C. trachomatis*-induced host cell lysis.

### FGF2 release into the media facilitates subsequent rounds of *C. trachomatis* infection

**Figure 9:**
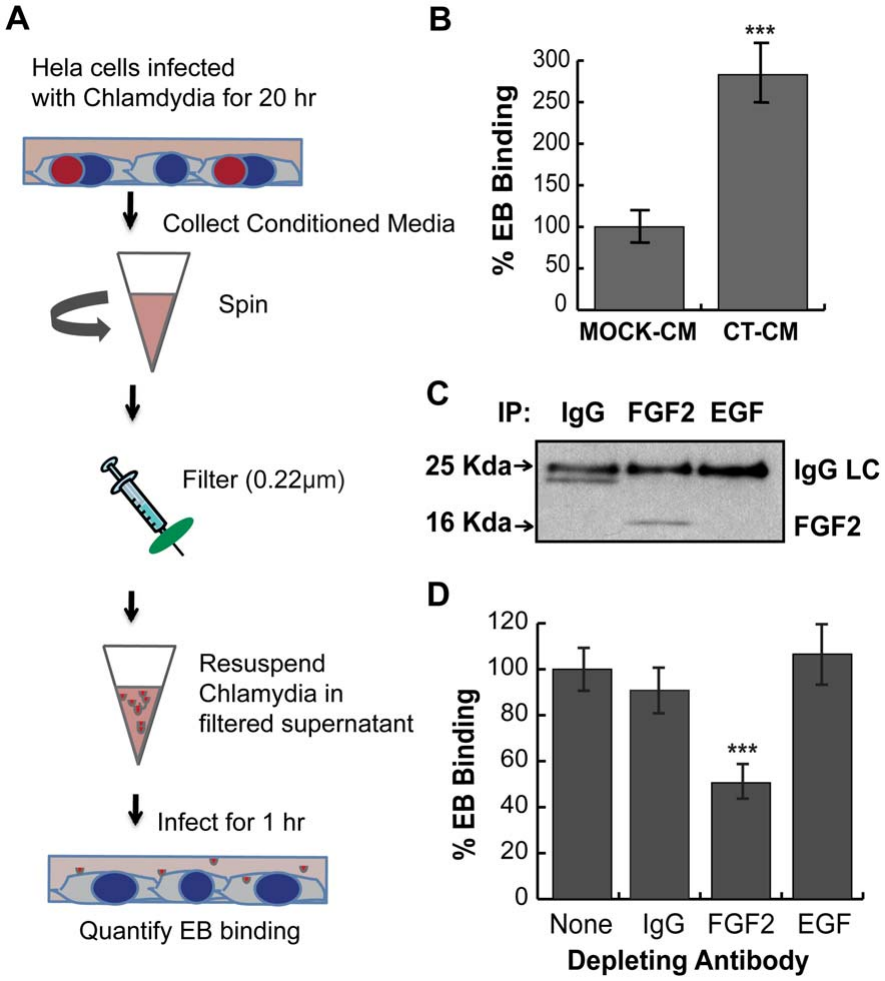
*C. trachomatis* L2-induced FGF2 facilitates secondary rounds of infection. (A) Schematic of experiment. HeLa cells were mock-infected or infected with *C. trachomatis* L2 for 20 hr in 2% FBS. Conditioned media from mock-infected cells (mock-CM) or from *C. trachomatis* L2 infected cells (CT-CM) were collected and filtered to remove residual bacteria and cell debris. (B) Renograffin-purified EBs were resuspended in mock-CM or CT-CM and binding to HeLa cells was measured at 1 hpi. Shown is the mean (± SEM) of three independent experiments. ***p<0.001 compared to Mock-CM. (C) Filtered CT-CM was immunoprecipitated with goat IgG (control), FGF2 antibody, or EGF antibody and immunoblotted with FGF2 antibody. The ∼24 kDa band in the IgG sample likely represents a breakdown product of IgG light chain (LC). (D) Renograffin-purified EBs were resuspended with CT-CM that had been immunodepleted (see Panel C) with the indicated antibody and binding to HeLa cells at 1 hpi was quantified. The values are normalized to EB binding in CT-CM without antibody incubation. Shown is the mean (± SEM) of three independent experiments. ***p<0.001 compared to no antibody depletion.

Since *C. trachomatis* infection stimulates FGF2 production and release from the host cells, we hypothesized that the increase in released FGF2 may facilitate subsequent rounds of *C. trachomatis* infection. We collected filtered conditioned media (CM) from *C. trachomatis*-infected HeLa cells at 20 hpi (CT-CM) or from mock-infected cells (mock-CM; see materials and methods and Fig 9A). The concentration of FGF2 in the CT-CM was ∼50 fold higher than mock-CM (data not shown). EBs resuspended in CT-CM enhanced EB binding 3-fold compared to mock-CM (p<0.001; Fig 9B). To determine whether FGF2 was responsible for the stimulatory effect of CT-CM, we immunodepleted FGF2 from the CT-CM using antibodies to FGF2. EB binding in the presence of FGF2-immunodepleted CT-CM was approximately 50% less efficient compared to CT-CM that had not been immunodepleted or that had been immunodepleted with a control goat IgG antibody or with antibodies to an unrelated growth factor, EGF (Figs 9C and 9D; P<0.005). To test for the efficiency of immunodepletion, we measured FGF2 by quantikine assay. Compared to untreated CT-CM, immunonodepletion with anti-FGF2 decreased the concentration of FGF2 by 60%, (data not shown). Together, these results suggest that *C. trachomatis* infection stimulates production and release of FGF2, which can then be co-opted by *C. trachomatis* to facilitate additional rounds of infection and bacterial spread.

### *C. trachomatis* serovar E also co-opts the FGF2 pathway

**Figure 10:**
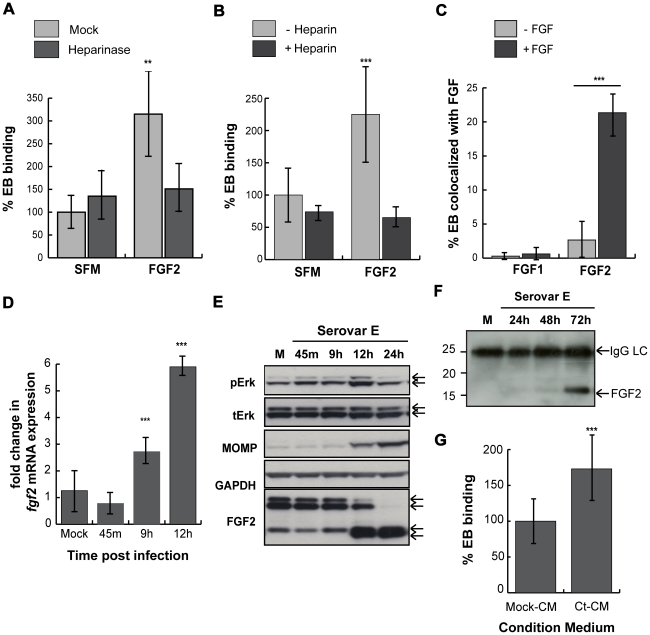
*C. trachomatis* serovar E co-opts the FGF2 pathway. (A) HeLa cells were treated with heparinase for 2 hr in SFM and infected with *C. trachomatis* serovar E either in SFM or in SFM supplemented with FGF2 (100 ng/mL) for 1 hr. Shown is the representative mean binding (± SEM) from three independent experiments. **p<0.01. (B) HeLa cells were serum starved for 2 hrs and then infected for 1 hr with *C. trachomatis* serovar E either in SFM or in SFM supplemented with FGF2 (100 ng/mL) in the presence or absence of heparin (1 mg/mL). Shown is the representative mean binding (± SEM) from three independent experiments. ***p<0.001 (C) Renograffin-purified serovar E EBs were incubated with 100 ng/mL of FGF1 or FGF2 in SFM containing 0.1% BSA for 1 hr at 37°C. The EB-FGF mixture was centrifuged onto coverslips, fixed, and stained with DAPI (to visualize the EBs) and FGF1 or FGF2 antibody. Co-localization of EBs with FGF1 or FGF2 was quantified from at least 8 different fields. The data are expressed as a mean percentage of bacteria associated with FGF (± SEM) compared to total bacteria. ***p<0.001. (D) Upper 2 panels: Erk1/2 activation in HeLa cells infected with *C. trachomatis* serovar E was examined by immunoblotting cell lyates with antibodies to pERK or to total ERK. The 42 and 44 kDa forms of ERK are indicated by the arrows. Middle panel: Cell lysates were immunoblotted with *C. trachomatis* MOMP antibody. Lower 2 panels: The change in cell-associated FGF2 isoforms in HeLa cells infected with *C. trachomatis* serovar E for the indicated times was assessed by immunoblotting cell lysates with antibodies to FGF2. GAPDH serves as a loading control. Arrows indicate the 24, 22.5/22, 18, and 16 kDa isoforms of FGF2. An abrupt change in FGF2 isoforms is noted at 10 hpi. (E) Total mRNA was isolated from HeLa cells infected with *C. trachomatis* serovar E for the indicated time and the fold change in *fgf2* mRNA relative to *gapdh* mRNA was measured by qRT-PCR. An increase in *fgf2* mRNA is detectable by 9 hpi and increases further at 12 hpi. ***p<0.001 compared to mock-infected cells. (F) HeLa cells were mock-infected for 72 hrs or infected with *C. trachomatis* serovar E in 5% FBS for 24, 48, or 72 hrs. Conditioned media were collected, filtered, immunoprecipitated with FGF2 antibody, and immunoblotted with FGF2 antibody. (G) Renograffin-purified serovar E EBs were resuspended in mock-CM or CM from serovar E infected (CT-CM). Binding to HeLa cells was measured at 1 hpi. Shown is the representative mean binding (± SEM) from three independent experiments. ***p<0.001 compared to mock-CM.

As serovar E binding has been reported to be variably sensitive to heparan sulfate [27], [57], [58], we investigated the role of the FGF2 pathway during epithelial cell infection. We first tested whether FGF2 can enhance serovar E binding to HeLa cells. Addition of FGF2 stimulated the binding of renograffin-purified serovar E to HeLa cells in SFM (Fig 10A and B). FGF2-enhanced binding was HSPG-dependent since it was inhibited by pretreatment of cells with heparinase (Fig 10A) or by competitive blocking with heparin (Fig 10B). Neither of these treatments significantly altered serovar E binding in the absence of exogenously added FGF2. Although renograffin-purified serovar E bound to purified FGF2 (Fig 10C), the binding was less efficient than serovar L2 (21% (Fig 10C) compared to 50% (Fig 3D). Depletion of endogenous FGF2 failed to significantly reduce serovar E binding to HeLa cells in the absence of serum (data not shown), suggesting that endogenous levels of FGF2 may not be sufficient to stimulate serovar E binding. Overall, the HSPG-dependence of serovar E appears qualitatively similar to that of serovar L2, although there are some quantitative differences.

We next examined whether serovar E infection induced FGF2 transcription, production, secretion, or processing. Serovar E infection of HeLa cells resulted in induction of *fgf2* transcription within 9 hrs (Fig 10D), biphasic activation of Erk1/2 phosphorylation (with peaks at 45 min and at 12 hrs; Fig 10E), and processing of the FGF2 isoforms beginning ∼12 hpi (Fig 10E). Conditioned medium harvested from serovar E infected cells at 72 hpi (a time at which host cell lysis commences) was enriched for the 16/18 kDa isoform of FGF2 (Fig 10F) and stimulated serovar E binding up to 70% compared to control conditioned medium (Fig 10G; p<0.001). Thus, at least two different serovars of *C. trachomatis* appear to be capable of co-opting the FGF2 pathway to facilitate bacterial spread.

## Discussion

**Figure 11:**
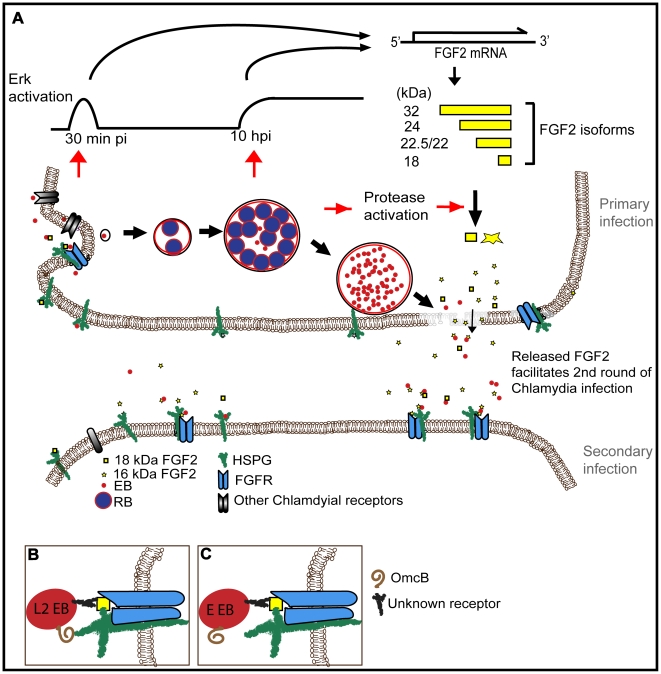
Working model for how *C. trachomatis* co-opts the FGF2 signaling pathway to enhance infection. (A) FGF2 binds to EBs and facilitates attachment and internalization through cell surface HSPG and FGFR. Both early (45 min pi, *C. trachomatis* replication independent) and late (10 hpi, *C. trachomatis* replication dependent) Erk1/2 activation induce *fgf2* transcription, resulting in an increase of all FGF2 isoforms. Subsequent intracellular replication of *C. trachomatis* activates host cell proteases that lead to the processing and/or loss of the HMW FGF2 isoforms and increased amounts of the 16 and 18 kDa isoforms. Upon host cell lysis, EBs, along with the 16 and 18 kDa FGF2 isoforms, are released. Released FGF2 binds to EBs and serves as a bridging molecule to enhance subsequent rounds of *C. trachomatis* binding and entry. This positive feedback loop between *C. trachomatis* and FGF2 may enhance the efficiency and spread of subsequent rounds of infection. (B) L2-FGF2 binding may involve synergistic interactions with OmcB-HSPG interactions. (C) The HSPG-binding domain of OmcB of serovar E is non-functional.

The molecular details of *Chlamydia trachomatis* binding, entry, and spread are incompletely understood. HSPGs are thought to play a role in the initial binding interactions. Since cell surface HSPGs facilitate the interactions of many growth factors with their receptors, we investigated the role of HSPG-dependent growth factors in *C. trachomatis* infection. Here, we report the novel finding that FGF2 is necessary and sufficient to enhance *C. trachomatis* binding to host cells in an HSPG-dependent manner. Unexpectedly, we found that FGF2 binds directly to EBs, where it may function as a bridging molecule to facilitate interactions of EBs with FGFR on the cell surface. Upon EB binding, FGFR is activated locally and contributes to bacterial uptake into non-phagocytic cells. We show that *C. trachomatis* infection stimulates *fgf2* transcription and enhances production and release of FGF2 through a pathway that requires bacterial protein synthesis and activation of Erk1/2 signaling but that is independent of FGFR activation. Intracellular replication of the bacteria results in host proteosome-mediated degradation of the HMW isoforms of FGF2 and increased amounts and release of the LMW isoforms. Finally, we demonstrate the *in vivo* relevance of these findings by showing that conditioned medium from *C. trachomatis* infected cells is enriched for FGF2 and that this accounts for its ability to enhance *C. trachomatis* infectivity in additional rounds of infection. Together, these results demonstrate that *C. trachomatis* utilizes multiple mechanisms to co-opt the host cell FGF2 pathway to enhance bacterial infection and spread (Fig 11A).

By several criteria, we found that the binding of FGF2 to EBs appears to be quite specific. We postulate that FGF2 functions as a bridging molecule, by binding simultaneously to EB surface proteins and to HSPGs and/or FGFR on the host cell (Fig 11B). Co-localization of FGF2 with purified EBs was not diminished by pre-treatment of EBs with heparinase, suggesting that FGF2 binding to EBs was not mediated by HSPGs. However, EB-FGF2 binding may involve synergistic interactions with OmcB, a cysteine rich outer membrane protein found in most chlamydial species that contains a HS binding domain and mediates attachment to HSPGs [59]. We further show that a consequence of FGF2 binding to EBs is that activated FGFR and FRS2α are recruited to the site of bacterial binding, facilitating uptake. Activation of FGFR, however, is not required for the Chlamydia-induced upregulation of *fgf2* transcription, production, processing, and release.

In previous work, we have shown that phospho-PDGFR co-localizes with bound EBs, but other growth factor receptors, such as EGFR, are not recruited [5], suggesting selectivity and specificity in growth factor receptor recruitment. Although PDGFR signaling has been shown to stimulate FGFR under some conditions, we did not find evidence for cross-talk in the setting of *C. trachomatis*-induced activation of FGFR in the absence of serum. However, using informative pharmacologic inhibitors, we found evidence that the PDGFR and FGFR pathways may function redundantly in *C. trachomatis* entry. Growth factor signaling may also be important at steps downstream of entry, for example by providing pro-survival signals for the host cell [60].

We found that *C. trachomatis* infection upregulates FGF2 transcription, production, and secretion. FGF2 transcription and production were upregulated within the first 12 hpi and continued for at least 24 hpi. This process was independent of FGFR activation, but involved biphasic activation of Erk1/2 kinases. Early Erk1/2 activation was independent of de novo bacterial protein synthesis. We speculate that the first wave of Erk1/2 activation may involve TARP, a chlamydial type III secreted protein that is present in EBs and then injected into the host cell cytoplasm upon bacterial binding. TARP has recently been shown to bind to SHC1 [42], a Src homology-2 domain containing protein that is recruited to and phosphorylated by FGFR (and EGFR) upon its activation and that subsequently mediates Erk1/2 activation [61], [62], [63]. Thus, recruitment and activation of FGFR may facilitate or synergize with TARP and other chlamydial factors to activate Erk1/2 as well as to enhance bacterial internalization. Indeed, the failure of the FGFR inhibitor PD173074 to completely block *C. trachomatis*-induced Erk1/2 activation may result from the redundant involvement of chlamydial factors, such as TARP, together with the activation of FGFR. The second peak of Erk1/2 activation required active bacterial protein synthesis. This finding suggests either that a de novo synthesized chlamydial protein (as opposed to an immediate early protein such as TARP) is secreted from the vacuole to activate the Erk pathway, or that Erk is activated in response to vacuolar and/or bacterial intracellular growth. In any case, we conclude that the two waves of Erk1/2 activation, which occur through separate pathways, contribute to upregulation of FGF2 expression.

Our work also reveals that midway through the chlamydial intracellular life cycle, there is a loss of the HMW FGF2 isoforms and a concurrent increase in the LMW isoforms (16/18 kDa). The change in the spectrum of FGF2 proteins was independent of Erk1/2 activation but required bacterial intracellular growth. We favor the idea that the HMW forms are degraded rather than processed into the 16 and/or 18 kDa form. The molecular identity of the 16 kDa isoform is currently under investigation, but may represent a previously reported pepstatin-sensitive acid proteinase cleavage product [19].

We considered several possible mechanisms for the change in FGF2 isoforms. First, the change in isoforms could result from a shift in the translation initiation sites. However, a modified pulse-chase experiment, in which we followed the isoform distribution after inhibiting host protein synthesis at 6–12 hpi with cycloheximide, demonstrated that the change in FGF2 isoforms still occurred, eliminating this possibility. Second, we tested whether the *Chlamydia* protease CPAF might be responsible for degrading or processing the FGF2 isoforms, but in vitro experiments using recombinant CPAF ruled out this notion. Third, and most likely, the change in FGF2 isoforms may be a consequence of *C. trachomatis*-induced activation of a host protease, as pretreatment with lactacystin or MG132 prevented the isoform change. In hematopoietic cells, thrombin has been reported to process the HMW FGF2 isoforms into an 18 kDa species [64], though this process seems less likely in epithelial cells that lack thrombin. However, it is possible that an as yet-identified bacterial-encoded protease could account for the processing.

Finally, we demonstrate that by enhancing secondary rounds of infection, *C. trachomatis*-induced up-regulation of FGF2 is physiologically important. Conditioned media from *C. trachomatis*-infected cells (CT-CM) stimulated EB binding. Two pieces of evidence provide support that FGF2 contributed to the activity of the CT-CM. First, there was an increase in FGF2 levels in CT-CM compared to CM isolated from mock-infected cells. Second, immunodepletion of FGF2 from the CT-CM decreased its ability to stimulate EB binding, whereas depletion with a control antibody or an irrelevant antibody was without effect. In addition to stimulating EB binding, we speculate that FGF2 production enhances secondary rounds of infection by its prosurvival activity.

We found both similarities and differences in the HSPG-dependence and modulation of FGF2 signaling of serovar E compared to serovar L2. As observed with L2, serovar E binding to HeLa cells was stimulated by FGF2 in an HSPG-dependent manner but was not affected by depletion of host cell FGF2. Serovar E bound to FGF2 in vitro, though perhaps less avidly. It is intriguing to speculate that the absence of a functional heparan sulfate binding domain in the OmcB surface protein of serovar E [59] may explain in part the decreased FGF2 binding (Fig 11C). Nonetheless, serovar E stimulated transcription, production, and processing of FGF2. Together these results suggest that serovar E activates the Erk pathway and FGF2 production similarly to serovar L2 and that it may utilize FGF2/HSPG-dependent pathway for binding. In the future, it will be interesting to determine whether FGFR signaling is activated upon serovar E binding.

Modulation of growth factor expression or distribution is an emerging theme in bacterial infections. *Neisseria gonorrhoeae* infection induces expression, processing and release of amphiregulin, an epidermal growth factor (EGF) family member that is anti-apoptotic [65]. *H. pylori* infection stimulates HB-EGF production, which may contribute to cancer progression [66]. *C. pneumoniae* infection of cultured endothelial cells has been reported to increase FGF2 and PDGF production, which may be responsible for smooth muscle cell proliferation and intimal thickening in aortic tissues, and could account for its potential association with atherosclerosis [67].

In summary, our results demonstrate that *C. trachomatis* co-opts FGF2 to enhance infection and bacterial spread (Fig 11A). Activation of the Erk1/2 pathway, either at the time of binding and entry or during subsequent intracellular growth, leads to increased *fgf2* transcription and production. In addition, intracellular growth activates host protease(s), resulting in alterations in the distribution of FGF2 isoforms and enhanced release of the secreted forms during host cell lysis. The released FGF2 serves as a bridging molecule to facilitate subsequent rounds of binding, entry, and intracellular development. This positive feedback loop amplifies secondary infection as well as promoting efficient bacterial spread. FGF2 may play additional roles in the pathogenesis of chlamydial infection, by potentiating the inflammatory response, by inhibiting apoptosis, or by modulating gene expression [68], [69], [70]. In the future, it will be interesting to determine whether FGF2 contributes to pelvic inflammatory disease and whether other human adapted chlamdyial species, such as *C. pneumoniae,* utilize FGF2 to enhance infection.

## Materials and Methods

### Reagents

Recombinant human FGF1 and FGF2 were purchased from Invitrogen and Leinko Technology. FGF10, PDGF, EGF, HB-EGF, VEGF were purchased from R&D systems. Heparin, Heparinase Cycloheximide, and MG132 (Z-Leu-Leu-Leu-al) were purchased from Sigma-Aldrich. PD173074 and AG1296 were purchased form Stemgent and Calbiochem, respectively. Carboxyfluorescein FLICA kit was purchased from ImmunocChemistry Technology. Caspase 1 inhibitors, YVAD and WEHD were purchased from Biovision and R&D systems, respectively. Chloramphenicol was purchased from Allstar. shRNA constructs specific for FGF2 or GFP were obtained from OriGene Technology. Quantikine kit used to measure FGF2 concentration was purchased from R&D systems. MEK inhibitor U0126 and the Cytotox 96 non-radioactive cytotoxicity assay kit used to measure LDH activity were purchased from Promega. QIAshredder, RNeasy kit, RNase-free DNase, and cDNA synthesis kit were purchased from Qiagen. SYBR GreenER qPCR SuperMix was purchased from Invitrogen.

Antibodies were obtained from the following sources: mouse anti-*Chlamydia* FITC conjugate from Meridian Diagnostics; goat anti-*C. trachomatis* MOMP and rabbit anti-*Chlamydia* LPS from Fitzgerald; mouse anti-GAPDH from Chemicon; mouse anti-FGFR1, mouse anti-FGFR2, rabbit anti-phospho-FGFR (Y653/654), normal goat IgG, goat anti-FGF2, and goat anti-EGF from R&D systems; rabbit anti-FRS2α from Santa Cruz Biotechnology; rabbit anti-phospho FRS2α (Y436), rabbit anti-Erk1/2, and mouse anti-phospho Erk1/2 (Y202/Y204) from Cell signaling technology; goat anti-human EGF from R&D systems; mouse anti-vimentin from Sigma-Aldrich; rabbit anti-caspase-1 from Biovision; HSPG-10E4 antibody from Seikagaku corp.; HRP-rabbit anti-goat IgG from Zymed; HRP-goat anti-rabbit IgG and goat anti-mouse IgG HRP from Amersham Biosciences; all fluorescently labeled secondary antibodies and phalloidin from Molecular Probes.

### Cell culture and *C. trachomatis* propagation

HeLa 229 cells and L929 cells were obtained from ATCC and passaged as previously described [71]. H292 cells were a gift from Dr. Lemjabbar-Alaoui (UCSF). *C. trachomatis* serovar L2 (LGV 434) was propagated in L929 cells grown in suspension culture and purified using a renograffin step-gradient as previously described [72]. *C. trachomatis* serovar E, a gift from Dr. Wyrick (East Tennessee State University), was propagated and purified as described previously with the following modifications [27]. *C. trachomatis* serovar E was grown in semiconfluent HeLa cells for 48 hrs in MEM supplemented with 10% FBS and cycloheximide (2 µg/mL). Cell monolayers were scraped with plunger and then sonicated. Cell debris was removed by centrifugation, and chlamydiae were purified using a renograffin step-gradient as previously described [72]. The final L2 or serovar E pellet was resuspended in sucrose phosphate buffer (SPG; 5 mM glutamine, 0.2 M sucrose. 0.2 M phosphate buffer) and stored at −80°C.

### IF studies

IF was carried out as previously described [5]. For each set of experiments, the exposure times were identical for all images. Images were analyzed with Metamorph (Molecular devices) or with Adobe Photoshop CS4 to count nuclei, EBs, or vacuoles. For all image analysis, a minimum of 8 fields was analyzed per treatment. Data were compiled from at least 3 independent experiments unless it is specified.

To determine colocalization of phosphorylated FGFR or FRS2α with EBs, HeLa cells were grown on glass coverslips in 24-well plates and infected with *C. trachomatis* for 45 min. Cells were fixed in 4% paraformaldehyde (PFA), permeabilized with 0.2% Triton, blocked in 1% bovine serum albumin (BSA) in phosphate-buffered saline (PBS), and incubated with rabbit anti-phospho-FGFR or anti-phospho-FRS2α for 1 hr. Cells were washed with three times with PBS and then incubated for 1 hr with Alexa-488 conjugated secondary antibody and in some cases with fluorophore-conjugated phalloidin.

### Quantitation of *C. trachomatis* binding and vacuole formation

HeLa cells were grown on glass coverslips in 24-well plates in MEM containing 10% FBS overnight, and were infected with *C. trachomatis* at an MOI of 2–3 (for quantitation of vacuole formation) or 10 (for quantitation of EB binding) in the presence or absence of FBS for 1 hr. For quantitation of binding, unbound bacteria were removed by washing three times with PBS, and the infected cells were fixed with 4% PFA for 30 min. For quantitation of vacuole formation, PBS-washed cells were incubated in fresh MEM containing 10% FBS for 20 hr. The cells were fixed and were permeabilized with 0.2% Triton X-100 for 15 min, followed by blocking in 2% FBS/1% Fish Skin Gelatin in PBS for 30 min. Bound EBs or vacuoles were visualized by staining with anti-*Chlamydia* MOMP antibody followed by staining with Alexa-488 conjugated secondary antibody. The host cell was visualized by staining the actin cytoskeleton with phalloidin-Alexa 594. Images were acquired and analyzed as described above. Data was presented as number of cell-associated EBs per cell or number of vacuoles per cell.

To analyze the effect of growth factors on *C. trachomatis* binding, HeLa cells were serum starved for 2 hrs and then infected with *C. trachomatis* in SFM in the absence or presence of growth factors (100 ng/mL). Binding and vacuole formation were measured at 1 hpi and 20 hpi, respectively. For heparinase treatment, HeLa cells grown on glass coverslips in 24-well plates were incubated with 1 unit of heparinase (Sigma) in 0.5 mL of MEM containing 0.1% BSA at 37°C for 2 hr. Enzyme-treated cells were washed three times with PBS, infected with *C. trachomatis*, and then analyzed as described above. The efficacy of heparinase treatment was examined by IF staining with 10E4 antibody, which recognizes HW N-sulfation.

### Analysis of growth factor binding to purified EBs

FGF1 or FGF2 (100 ng/mL) was added to renograffin purified EBs (1×10^7^ IFU) suspended 1 mL of MEM containing BSA (0.1%), incubated with gentle agitation for 1 hr at 37°C, transferred to coverslips in 24-well plates, and centrifuged at 1000 rpm for 10 min. Unbound bacteria were removed by washing three times with PBS. EBs were visualized by staining with DAPI. FGF was visualized by staining with goat anti-FGF1 or anti-FGF2 antibody followed by staining with Alexa-488 conjugated secondary antibody. In some case, EBs (1×10^7^ IFU) was pretreated heparinase 1 unit in 0.5 mL of MEM containing 0.1% BSA at 37°C for 2 hr before incubating with FGF2. To examine the specificity of the *C. trachomatis*-FGF2 interaction, goat IgG or goat anti-FGF2 antibody (2 µg/mL) was added to the mixture of EBs-FGF2 and incubated for 1 hr rotating in 37°C. Data is presented as percentage of EB associated with FGF1 or FGF2 relative to total EB.

### shRNA depletion

HeLa cells grown in 6-well plates were transfected with the indicated shRNA according to manufacturer's protocol. At 48 hrs post transfection, the cells were trypsinized and reseeded onto glass coverslips in 24-well plates. At 72 hrs post transfection, cells were infected with *C. trachomatis* in SFM for 1 hr and then fixed. Lysates from shRNA-treated cells were immunoblotted with antibodies to FGF2 to determine the efficiency of FGF2 depletion.

### Immunoblot analysis

HeLa cells were lysed for 15 min on ice in Lysis Buffer (50 mM Tris HCl, pH 7.5, 150 mM NaCl, 1% Triton X-100, 1 mM EDTA, 50 mM NaF, 1% sodium deoxycholate, 0.1% SDS, 1 mM sodium orthovanadate, 0.1 mM okadaic acid, and Complete protease inhibitors (Roche Diagnostics)). Cell lysates were collected, centrifuged at 20,800 g for 15 min to remove cell debris, and the supernatant was boiled in NuPage 4X LDS sample buffer (Invitrogen) with 100 mM DTT for 10 min. Proteins in the supernatant were separated on 10% NuPAGE Novex Bis-Tris gels (Invitrogen) and transferred to 0.45 mm Trans-blot nitrocellulose membranes (BioRad Laboratories). Membranes were rinsed in water, followed by blocking with 3% milk (Upstate) in Tris-buffered saline (TBS) for 1 hr. Each membrane was incubated with the indicated antibody in 3% milk in TBS with 0.02% Tween-20 (TBST) overnight at 4°C, followed by an incubation with the appropriate HRP-conjugated antibodies for 1 hr. HRP-conjugated antibodies were detected by ECL (Amersham Biosciences) according to the manufacturer's protocol. For quantification, band intensity was analyzed using Metamorph image analysis software (Molecular devices, Sunnyvale, CA).

### Inside-out staining

Inside-out staining was performed as described previously [5] with the following modifications. HeLa cells grown overnight on glass coverslips in 24-well plates were infected with *C. trachomatis* for 1 hr at 37°C. Cells were washed three times with PBS to remove unbound bacteria and then fixed in 1% PFA for 15 min, conditions under which the host cell plasma membrane is not permeabilized. After fixation, cells were blocked in 2% FBS/1% FSG/PBS for 30 min and then incubated with goat anti-MOMP antibody for 1 hr followed by incubation with donkey anti-goat Alexa 488 antibody to stain external EBs. Cells were then permeabilized with 0.2% Triton X-100 for 15 min, blocked in 2% FBS/1% FSG/PBS for 30 min again, and incubated with rabbit anti-*Chlamydia* LPS antibody followed by incubation with goat anti-rabbit Alexa 594 antibody to stain both intracellular and extracellular EBs. The host cells were visualized by staining the actin cytoskeleton with phalloidin-Alexa 350. All images were acquired and analyzed as described in Immunofluorescence studies. The percent efficiency of internalization was calculated as follows: (number of total cell associated EB-number of extracellular EB)/(number of total cell associated EB)×100.

### *In vitro* recombinant CPAF studies

HeLa cells grown in T-75 flask at ∼100% confluency (8.4×10^6^ cells) were harvested and lysed in 200 µL 50 mM Tris buffer (pH 7.5) with 1X protease inhibitory cocktail (Roche) and 5 µM PMSF. Cell lysates were sonicated gently and centrifuged at 10,600 X *g* for 5 min at 4°C. Supernatants were split into four samples (∼50 µL each). Purified recombinant CPAF (a kind gift of Dr. Raphael Valdivia) was added to three samples and incubated for 30 min at 4°C, 10 min at 37°C, or 30 min at 37°C. Incubation without CPAF for 30 min at 37°C served as a negative control. Samples were boiled with NuPage LDS sample buffer (Invitrogen) and immunoblotted with antibodies to FGF2, vimentin, or GAPDH.

### Measuring FGF2 concentration and LDH activity

HeLa cells grown in 24-well plate were infected with *C. trachomatis* for 1 hr, washed, and then incubated in 0.5 mL media containing 2% FBS for the indicated times. At the end of incubation, *C. trachomatis*-conditioned media (CT-CM), control conditioned medium (Mock-CM), and cell lysates were collected. The supernatants were centrifuged at 1000 rpm to remove cell debris and filtered through a 0.22 µm filter to remove any EBs. The mock or L2 infected cells were washed once with PBS, lysed with 0.5 mL lysis buffer, centrifuged for 10 min at 20,800 X *g*, and then the supernatants were collected. FGF2 concentration in CT-CM and Mock-CM were measured using the Quantikine kit according to manufacturer's protocol. Total FGF2 represents the FGF2 concentration of the cell lysate and the supernatant. The percentage of released FGF2 was calculated as released FGF2/total FGF2.

Portions of the supernatant and cell lysate were diluted and quantified for LDH activity using the Cytotox-96 kit according to manufacturer's protocol. Total LDH activity (cell associated plus released) or released LDH activity was calculated as described above.

### Caspase-1 activation

*C. trachomatis* infected HeLa cells were labeled with FAM-YVAD-fmk caspase-1 FLICA kit according to the manufacturer's protocol at 12, 18, or 24 hpi. Hoechst dye was used to stain the host cell nuclei during the last 5 min of incubation of FLICA reagents. Excess dye was removed by washing three times with PBS and IF images of live cells were taken immediately. The percentage of FLICA positive cells relative to the total cell number was calculated.

To assess the effect of inhibition of Caspase-1 activation on FGF2 release, *C. trachomatis*-infected HeLa cells were incubated with DMSO, YVAD (100 µM), or WEHD (100 µM) from 12 hpi to 24 hpi. The ratios of FGF2/LDH activity in the supernatant in the presence of inhibitors were measured as described above and compared to the ratio in control (DMSO-treated) cells.

### Isolation of RNA and DNA, cDNA synthesis, and Real Time PCR

RNAs from uninfected and *Chlamydia*-infected HeLa cells were isolated using the QIAshredder and the RNeasy kit according to the manufacturer's instructions. RNA was treated with RNase-free DNase according to the manufacturer's instruction. RNA concentrations were measured using Nanodrop Spectrophotometer (Thermo-scientific). One µg of RNA was reverse transcribed using cDNA synthesis kit in a 20 µL reaction. Quantitative PCR (qPCR) was performed with 2 µL of the cDNA preparation using SYBR GreenER qPCR SuperMix in a 25 µL reaction using DNA Engine Opticon-2 Real-Time PCR Detection System in the Opticon-2 Real-Time Cycler (BioRad). Primers for human *gapdh* were 5′-CTTCTCTGATGAGGCCCAAG-3′ forward and 5′-GCAGCAAACTGGAAAGGAAG-3′ reverse. Primers for human *fgf2* were 5′-CGTGCTATGAAGGAAGATGGA3′ forward and 5′-TGCCCAGTTCGTTTCAGT-3′ reverse. qPCR included initial denaturation at 94°C for 10 min, followed by 35 cycles of 94°C for 10 s, 53°C for 15 s, 72°C for 20 s, 72°C for 1 s and then 72°C for 10 min followed by a dissociation curve every 0.5°C from 55°C to 95°C. In a dissociation curve, a single peak was confirmed in each of the amplified sequences. For the quantification of *fgf2* expression relative to *gapdh* in different samples, the threshold cycle (Ct) values of targets were expressed as 2^−ΔΔCt^ (fold) as described previously [73]. Each sample was additionally amplified without reverse transcription reaction to confirm the absence of contaminating DNA in the RNA sample.

For quantitative PCR of *Chlamydia groEL* relative to human *gapdh*, DNA from infected and uninfected Hela cells was isolated using the Gentra Puregene kit (Qiagen) according to the manufacturer's instructions. qPCR was performed with 100 ng of RNase treated DNA. The primers for *Chlamydia groEL* were 5′-GCTCATCTTCATTAGTCAACATTGG-3′ forward and 5′-CTCTCTGGTGGAGTAGCAGTCATT-3′ reverse. The qPCR cycle included 2 min at 50°C, 10 min at 94°C, followed by 35 cycles of 94°C for 15 s, 57°C for 45 s, and 72°C for 20 s. After the cycle, it was followed by a dissociation curve every 0.5°C from 55°C to 95°C. Relative gene copies of *groEL* to *gapdh* was expressed as 2^−ΔΔCt^ (fold) as described above.

### Conditioned media

HeLa cells grown in 6-well plates were mock-infected or infected with *C. trachomatis* for 1 hr. Unbound bacteria were removed by washing three times with PBS, and infected cells were incubated in 1 mL of media containing 2% FBS for 20 hrs. At the end of incubation, the conditioned media from mock-infected cells (Mock-CM) or *C. trachomatis* infected cells (CT-CM) was collected and centrifuged 5 min at 240 X *g*. The CM was passed through a 0.22 µm filter to remove any residual EBs. EBs were resuspended in filtered Mock-CM or CT-CM and vacuole formation was quantified at 18 hpi.

For immunoprecipitation of FGF2, the filtered MOCK-CM or CT-CM was incubated for 2 hrs with 4 µg of anti-FGF2 goat IgG preconjugated to 50 µL of Protein G Sepharose TM 4 Fast Flow (GE HealthCare) at 4°C. Immunoprecipitates were recovered by centrifugation at 1 min at 1000 rpm. The immunoprecipitates were boiled in LDS sample buffer containing 100 mM dithiothreitol and then subjected to immunoblotting to detect FGF2.

For immunodepletion of FGF2, the filtered CT-CM was depleted with normal goat IgG (R&D system), anti-FGF2 goat IgG, or anti-EGF goat IgG preconjugated to Protein G Sepharose TM 4 Fast Flow (GE HealthCare). Supernatants were collected after centrifugation and used for further infection. Immunoprecipitates were recovered and subjected to immunoblotting as mentioned above to ensure FGF2 or EGF depletion.

### Statistical analysis

Data represented the mean ± standard error of at least experiments. Statistical analysis was performed using the software program InStat. The significance between groups was determined by ANOVA. p<0.05 was considered to be statistically significant.

### Gene IDs

FGF1 (2246), FGF2 (2247), FGF10 (2255), FGFR1 (2260), FGFR2 (2263), FGFR3 (2261), FGFR4 (2264), FRS2α (10818), ERK-1 (5595), ERK-2 (26413), PDGFR-β (5159), PDGF-B (18591), EGF (1950), HB-EGF (1839), and VEGF (7422).

## Supporting Information

Figure S1 **FGF2 stimulates** ***C. trachomatis*** **L2 binding in a do\se-dependent, saturable manner.** (A) HeLa cells were serum starved for 2 hrs and then infected with *C. trachomatis* L2 in SFM supplemented with the indicated concentration of FGF2 for 1 hr. Binding was quantified at 1 hpi. Shown is the mean number of EBs bound per cell (± SEM), representative of two independent experiments. (B) FGF2 stimulates *C. trachomatis* vacuole formation in H292 cells. H292 cells were serum starved for 2 hr and then infected with *C. trachomatis* L2 in SFM or in SFM supplemented with FGF2 (100 ng/mL). Vacuole formation was quantified at 20 hpi. Shown is the mean ± SEM, representative of two independent experiments. *** p<0.001
(TIF)

Figure S2 **Heparinase treatment of HeLa cells decreased surface staining with an anti-heparan sulfate (10E4) antibody.** (A) Hela cells pretreated with heparinase (1 unit) for 2 hrs were fixed and stained with anti-heparan sulfate (10E4) Ab (green) and DAPI (blue). Shown are 4 representative fields. (B) Total Heparan sulfate signal per cell was measured using Metamorph image analysis software, and is expressed as arbitrary units (AU). Shown is the average value of at least 6 different fields (± SEM). *p<0.05
(TIF)

Figure S3 ***C. trachomatis*** **L2 infection does not stimulates** ***fgf1*** **transcription.** Total RNA was isolated from the HeLa cells infected with *C. trachomatis* at the indicated times post infection. *fgf1* mRNA was assessed by qRT-PCR and normalized to *gapdh* mRNA. Data shown is representative of two independent experiments.
(TIF)

Figure S4 **Erk1/2 activation is not necessary for** ***C. trachomatis*** **L2 binding, internalization, or replication.** (A,B) HeLa cells were infected with *C. trachomatis* L2 for 1 hr in FBS-containing media supplemented with DMSO or U0126 (10 µM). Bound or internalized EBs were quantified by inside-out staining as described in Materials and Methods. Internalization efficiency is expressed as a mean percentage ± SEM of internalized EBs compared to total cell associated EBs. Data shown is representative of three independent experiments. (C) HeLa cells were infected with *C. trachomatis* L2 for 12 hr. As indicated, U0126 (U, 10 uM) was present for the first 3 hpi (I+U 0–3 h) or the last 4 hpi (I+U 8–12 h). Total DNA was isolated and *C. trachomatis* L2 replication was assessed by qPCR of *groEL* relative to *gapdh* DNA.
(TIF)

Figure S5 **FGFR activation contributes to** ***C. trachomatis*** **L2-induced early Erk1/2 activation but is not required for up regulation of** ***fgf2*** **transcription.** HeLa cells were infected with *C. trachomatis* L2 in SFM supplemented with DMSO (DM), PD173074 (PD; 200 nM), AG1296 (AG; 10 µM), or both (P/A). (A) Cell lysates were collected at 45 min pi and were immunoblotted with antibodies to phospho-Erk1/2, total Erk1/2, or GAPDH (loading control). Immunoblots are representative of three independent experiments. (B) HeLa cells were infected for 12 hrs. Inhibitors were present for the first 2 hrs. Total mRNA was isolated at 12 hpi and the fold change in *fgf2* mRNA relative to *gapdh* mRNA was measured by qRT-PCR. The results are normalized to uninfected cells (UI). Shown is the mean ± SEM representative of three independent experiments.
(TIF)

Figure S6 **Caspase-1 activation is not required for** ***C. trachomatis*** **L2** -**stimulated FGF2 release.** (A) HeLa cells were infected with *C. trachomatis* L2 for the indicated times. During the last hour of infection, cells were labeled with FAM-YVAD-fmk caspase-1 as described in the Materials and Methods. Shown is the percentage of FLICA positive cells among ∼5000 cells. (B) HeLa cells were infected with *C. trachomatis* L2 for 24 hrs in the presence of DMSO, YVAD or WEHD between 12–24 hpi. Secreted FGF2 was quantified in the media. Results are normalized to DMSO-treated cells. Shown is the mean ± SEM representative of 3 independent experiments.
(TIF)
