# Correction: *Chlamydia trachomatis* Co-opts the FGF2 Signaling Pathway to Enhance Infection

**DOI:** 10.1371/annotation/19deb814-0602-4909-8605-61ab6d79f85f

**Published:** 2013-08-06

**Authors:** Jung Hwa Kim, Shaobo Jiang, Cherilyn A. Elwell, Joanne N. Engel

The following should be included as the first sentence of the abstract:

“The strain designated Chlamydia trachomatis serovar L2 that was used for experiments in this paper is Chlamydia muridarum, a species closely related to C. trachomatis (and formerly termed the Mouse Pneumonitis strain of C. trachomatis). This conclusion was verified by deep sequencing and by PCR using species-specific primers. All data presented in the results section that refer to C. trachomatis should be interpreted as referring to C. muridarum. The authors have confirmed the identity of the C. trachomatis serovar E strain used in this study by PCR and sequencing of the ompA gene.”

The sentence at the bottom of page 10 should be corrected. It currently reads, “Thus, at least two different serovars of C. trachomatis appear to be capable of co-opting the FGF2 pathway to facilitate bacterial spread.” We would correct it to “Thus, at least two different species of Chlamydia appear to be capable of co-opting the FGF2 pathway to facilitate bacterial spread.”

