# Supplementary figures and images for: Chlamydia trachomatis Co-opts the FGF2 Signaling Pathway to Enhance Infection
Source: PLoS Pathog. 2011 Oct 6;7(10):e1002285. doi: 10.1371/journal.ppat.1002285 (PMC3188521; doi:10.1371/journal.ppat.1002285)

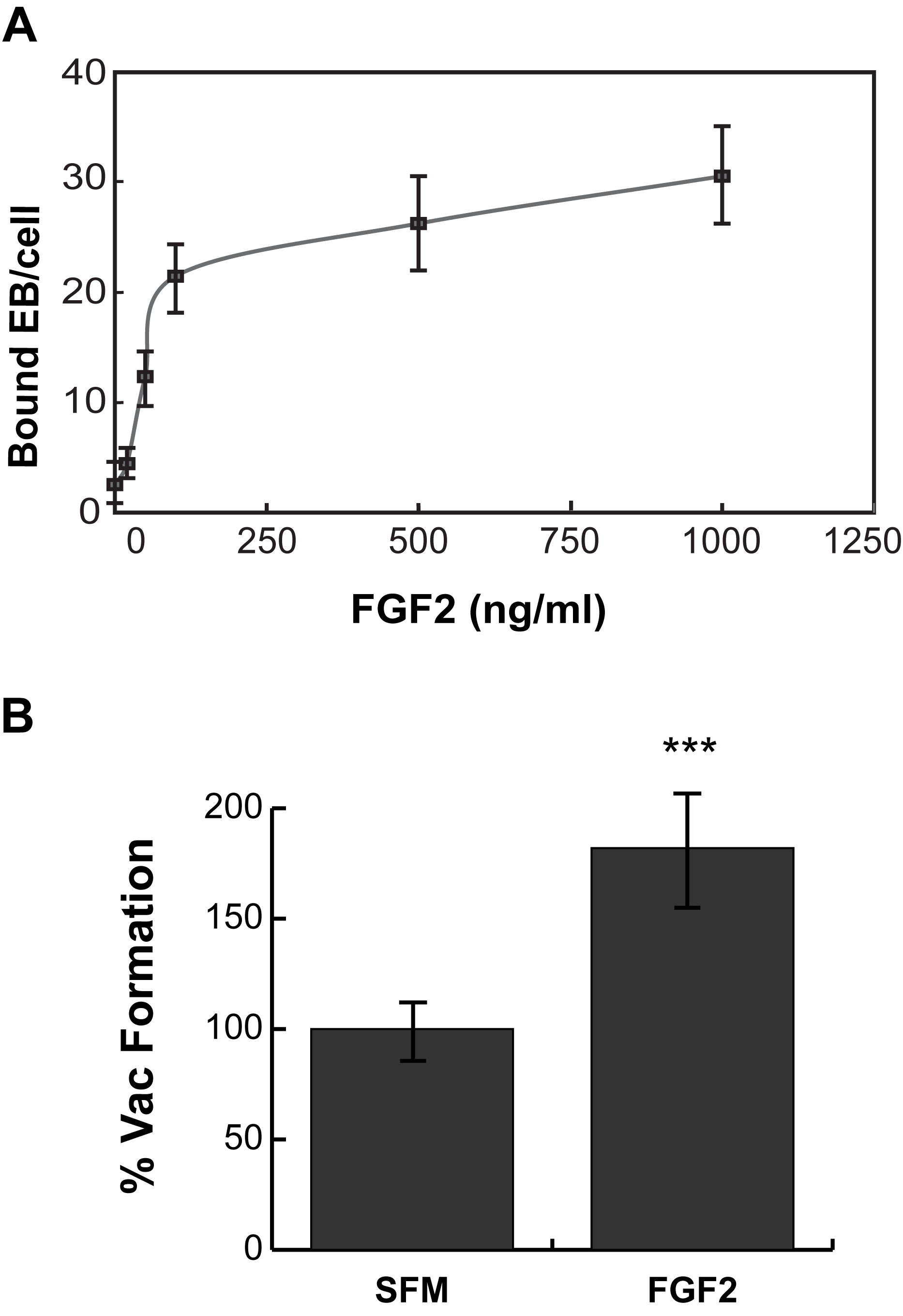

Supplement: Figure S1 — FGF2 stimulates C. trachomatis L2 binding in a do\se-dependent, saturable manner. (A) HeLa cells were serum starved for 2 hrs and then infected with C. trachomatis L2 in SFM supplemented with the indicated concentration of FGF2 for 1 hr. Binding was quantified at 1 hpi. Shown is the mean number of EBs bound per cell (± SEM), representative of two independent experiments. (B) FGF2 stimulates C. trachomatis vacuole formation in H292 cells. H292 cells were serum starved for 2 hr and then infected with C. trachomatis L2 in SFM or in SFM supplemented with FGF2 (100 ng/mL). Vacuole formation was quantified at 20 hpi. Shown is the mean ± SEM, representative of two independent experiments. *** p<0.001 (TIF) [file ppat.1002285.s001.tif]

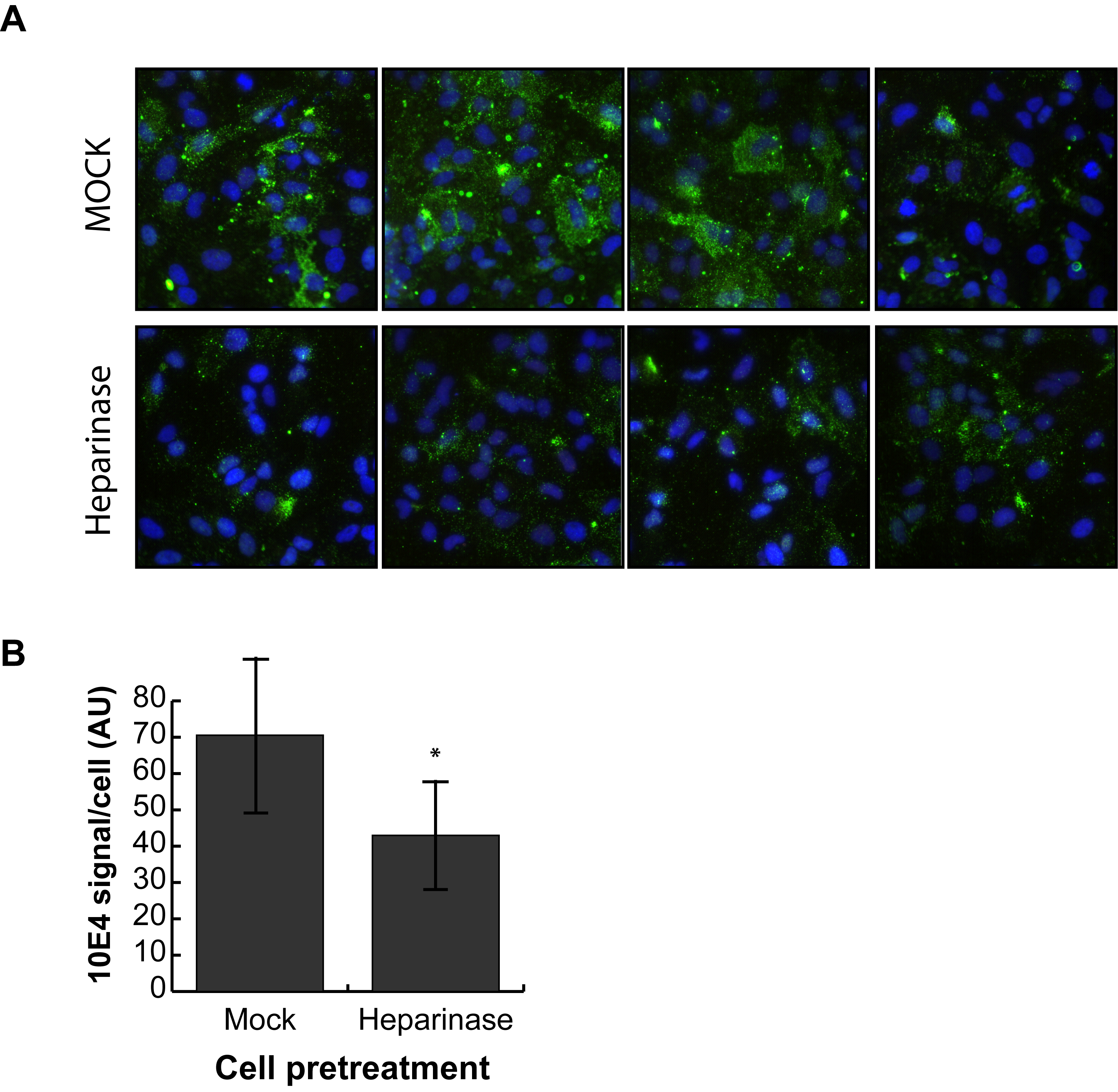

Supplement: Figure S2 — Heparinase treatment of HeLa cells decreased surface staining with an anti-heparan sulfate (10E4) antibody. (A) Hela cells pretreated with heparinase (1 unit) for 2 hrs were fixed and stained with anti-heparan sulfate (10E4) Ab (green) and DAPI (blue). Shown are 4 representative fields. (B) Total Heparan sulfate signal per cell was measured using Metamorph image analysis software, and is expressed as arbitrary units (AU). Shown is the average value of at least 6 different fields (± SEM). *p<0.05 (TIF) [file ppat.1002285.s002.tif]

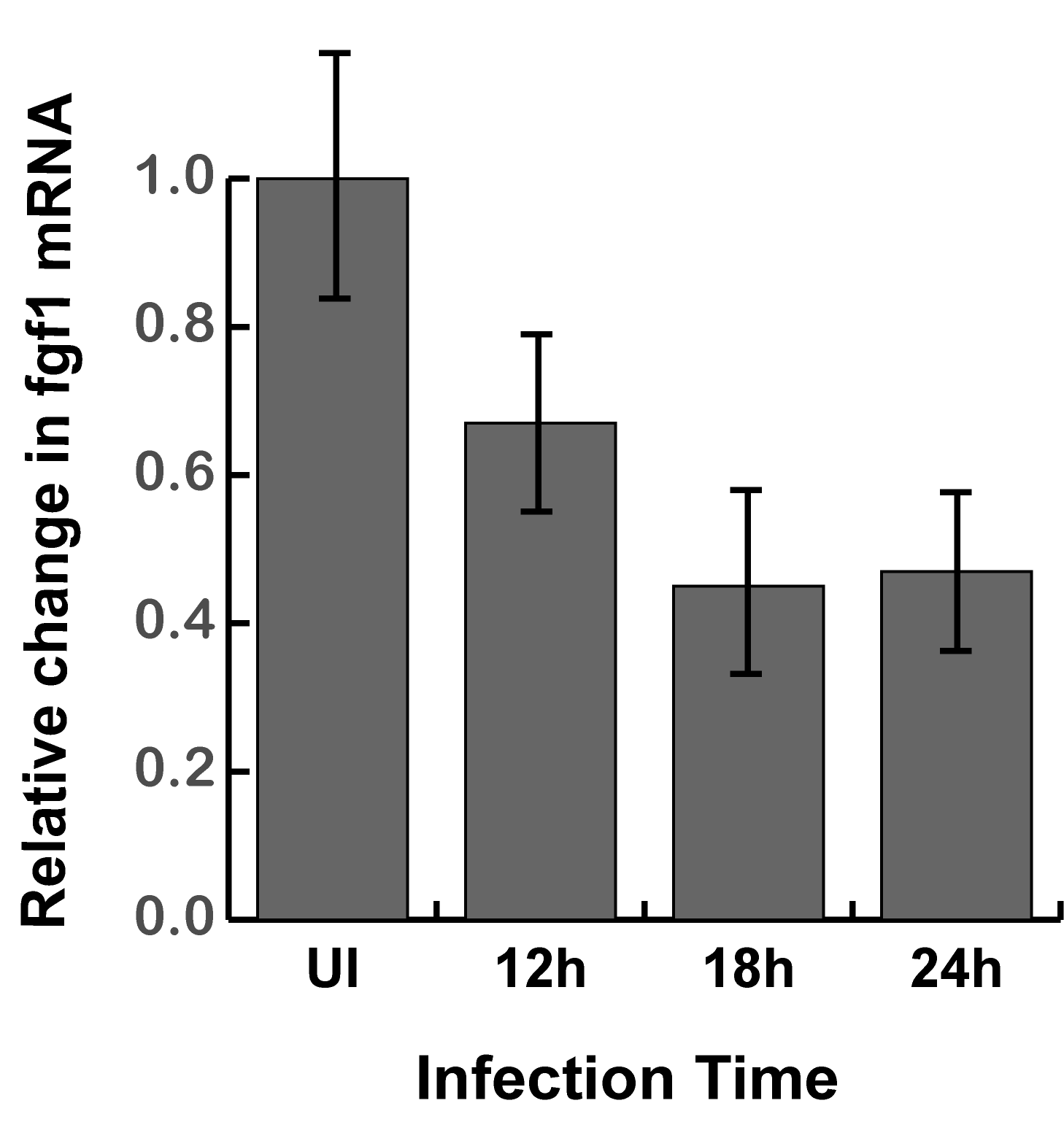

Supplement: Figure S3 — C. trachomatis L2 infection does not stimulates fgf1 transcription. Total RNA was isolated from the HeLa cells infected with C. trachomatis at the indicated times post infection. fgf1 mRNA was assessed by qRT-PCR and normalized to gapdh mRNA. Data shown is representative of two independent experiments. (TIF) [file ppat.1002285.s003.tif]

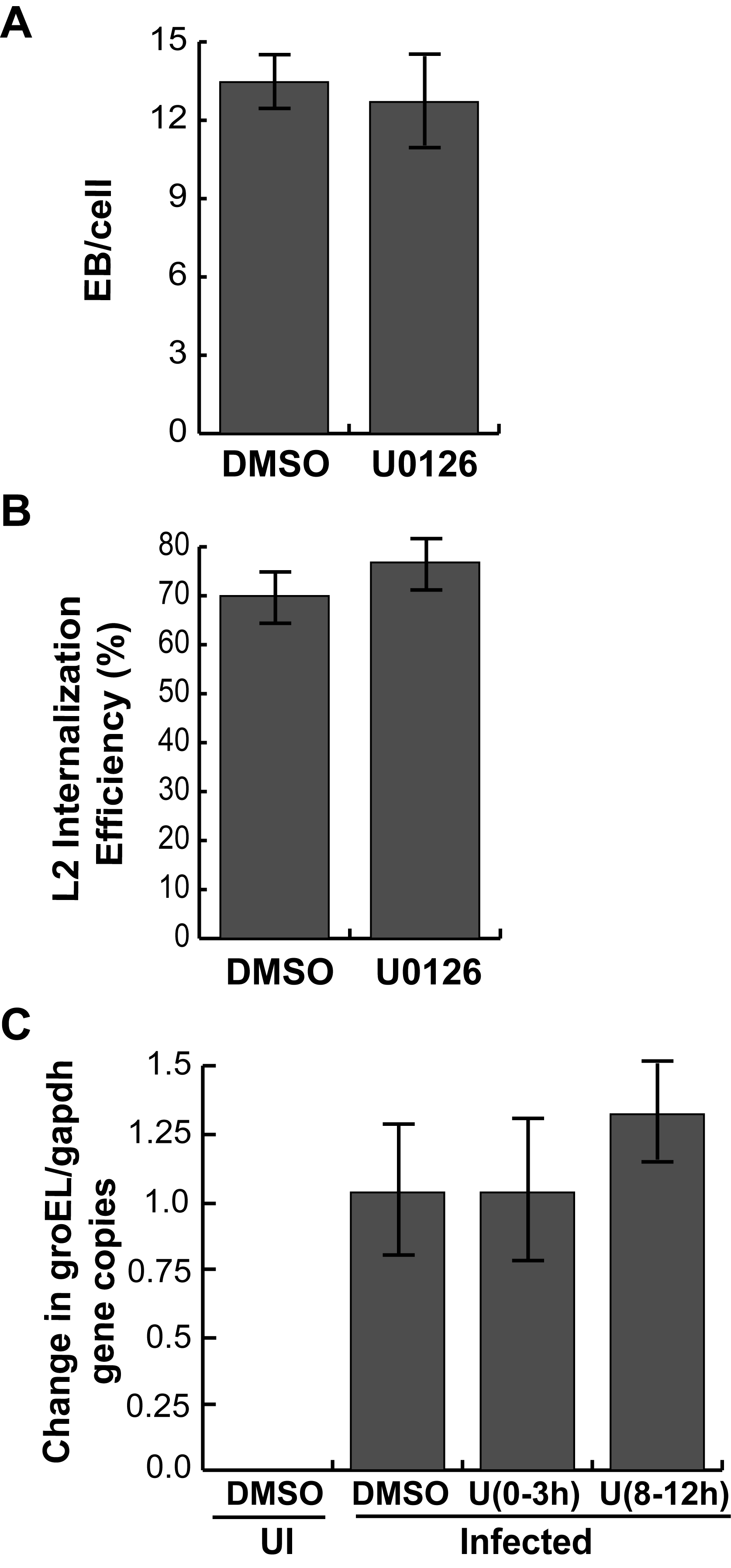

Supplement: Figure S4 — Erk1/2 activation is not necessary for C. trachomatis L2 binding, internalization, or replication. (A,B) HeLa cells were infected with C. trachomatis L2 for 1 hr in FBS-containing media supplemented with DMSO or U0126 (10 µM). Bound or internalized EBs were quantified by inside-out staining as described in Materials and Methods. Internalization efficiency is expressed as a mean percentage ± SEM of internalized EBs compared to total cell associated EBs. Data shown is representative of three independent experiments. (C) HeLa cells were infected with C. trachomatis L2 for 12 hr. As indicated, U0126 (U, 10 uM) was present for the first 3 hpi (I+U 0–3 h) or the last 4 hpi (I+U 8–12 h). Total DNA was isolated and C. trachomatis L2 replication was assessed by qPCR of groEL relative to gapdh DNA. (TIF) [file ppat.1002285.s004.tif]

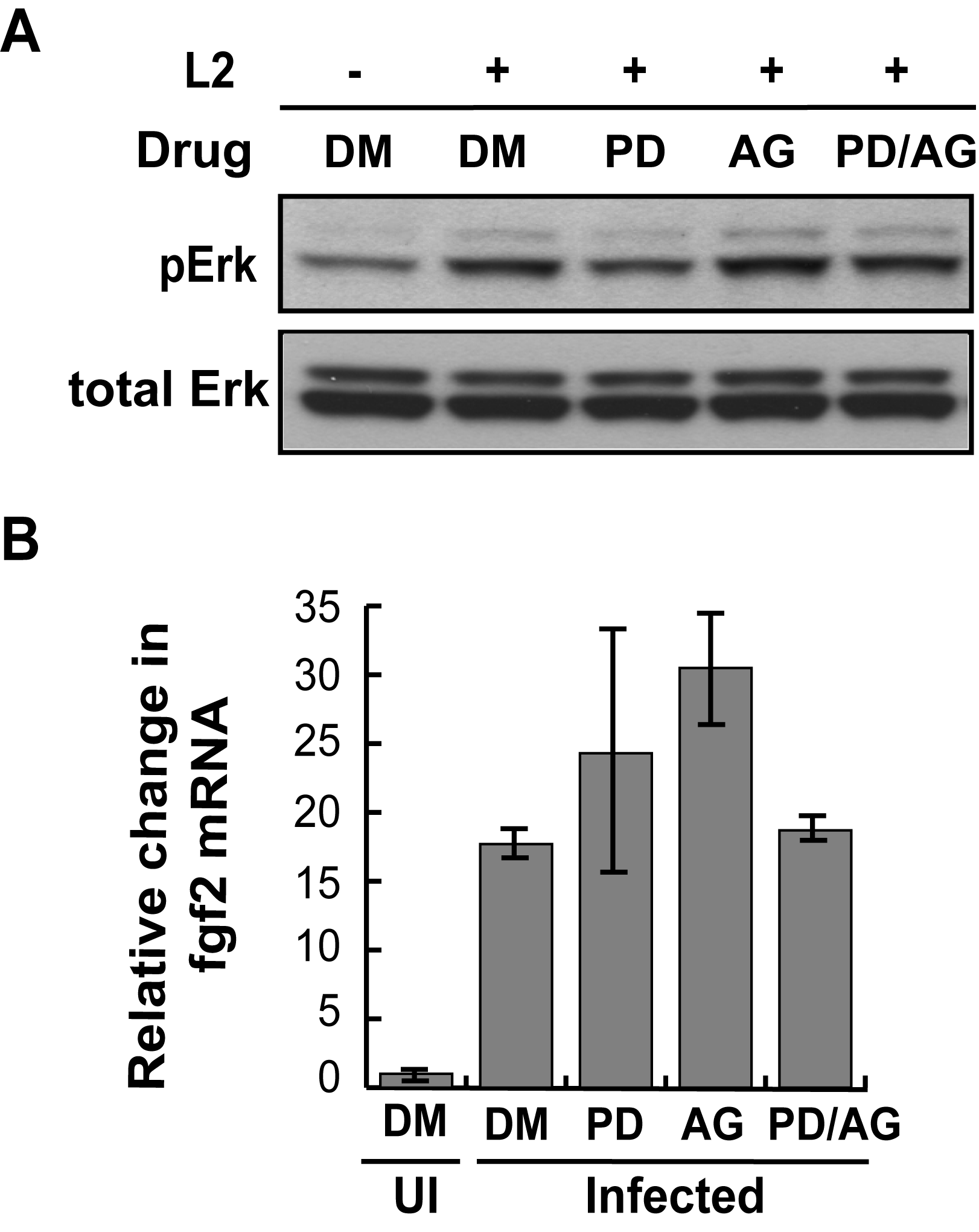

Supplement: Figure S5 — FGFR activation contributes to C. trachomatis L2-induced early Erk1/2 activation but is not required for up regulation of fgf2 transcription. HeLa cells were infected with C. trachomatis L2 in SFM supplemented with DMSO (DM), PD173074 (PD; 200 nM), AG1296 (AG; 10 µM), or both (P/A). (A) Cell lysates were collected at 45 min pi and were immunoblotted with antibodies to phospho-Erk1/2, total Erk1/2, or GAPDH (loading control). Immunoblots are representative of three independent experiments. (B) HeLa cells were infected for 12 hrs. Inhibitors were present for the first 2 hrs. Total mRNA was isolated at 12 hpi and the fold change in fgf2 mRNA relative to gapdh mRNA was measured by qRT-PCR. The results are normalized to uninfected cells (UI). Shown is the mean ± SEM representative of three independent experiments. (TIF) [file ppat.1002285.s005.tif]

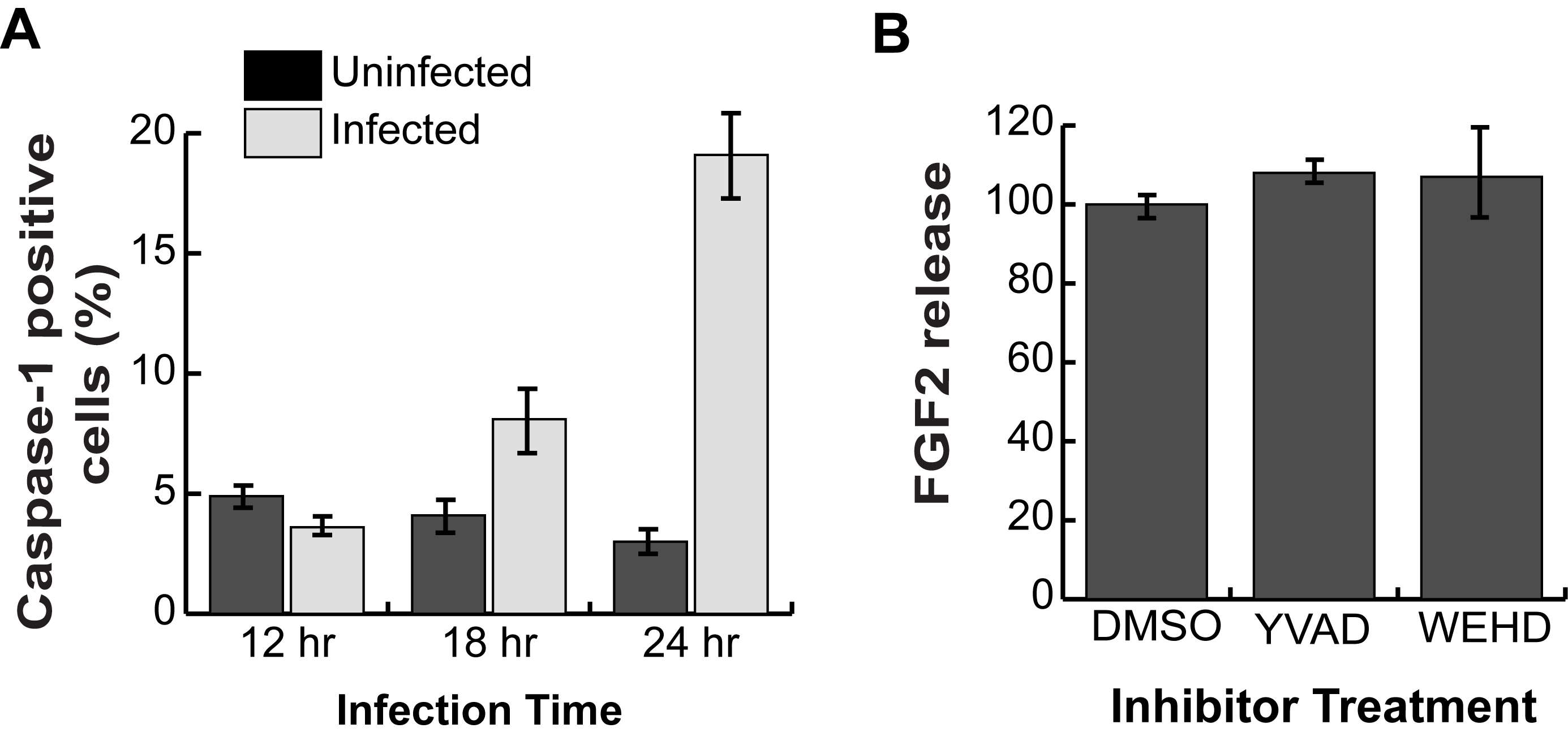

Supplement: Figure S6 — Caspase-1 activation is not required for C. trachomatis L2 -stimulated FGF2 release. (A) HeLa cells were infected with C. trachomatis L2 for the indicated times. During the last hour of infection, cells were labeled with FAM-YVAD-fmk caspase-1 as described in the Materials and Methods. Shown is the percentage of FLICA positive cells among ∼5000 cells. (B) HeLa cells were infected with C. trachomatis L2 for 24 hrs in the presence of DMSO, YVAD or WEHD between 12–24 hpi. Secreted FGF2 was quantified in the media. Results are normalized to DMSO-treated cells. Shown is the mean ± SEM representative of 3 independent experiments. (TIF) [file ppat.1002285.s006.tif]
